# Subjective reliving of past events is modulated by premotor–hippocampal coupling and bodily self-consciousness during event encoding

**DOI:** 10.1162/IMAG.a.1059

**Published:** 2025-12-15

**Authors:** Nathalie Heidi Meyer, Lucas Burget, Baptiste Gauthier, Jevita Potheegadoo, Juliette Boscheron, Olaf Blanke

**Affiliations:** Laboratory of Cognitive Neuroscience, Neuro-X Institute, Faculty of Life Sciences, Ecole Polytechnique Fédérale de Lausanne, Geneva, Switzerland; Clinical Research Unit, Neuchâtel Hospital Network, Neuchâtel, Switzerland; Department of Clinical Neurosciences, University Hospital Geneva, Geneva, Switzerland

**Keywords:** autonoetic consciousness, bodily self-consciousness, virtual reality, sense of reliving, hippocampus, general psychophysiological interaction

## Abstract

Autonoetic consciousness (ANC) enables individuals to relive past events by recalling sensory and emotional details tied to a specific place and time related to a past event. Next to ample evidence linking ANC to memory-related mechanisms, recent behavioral evidence suggests that ANC is modulated by sensorimotor and multisensory bodily mechanisms that are fundamental for bodily self-consciousness (BSC). However, the neural mechanisms of how BSC modulates ANC remain unclear. Based on previous work showing that activity in dorsal premotor cortex (dPMC) and hippocampus indexes BSC effects and its related sense of agency (SoA) in episodic memory, we here explored whether functional connectivity between dPMC and hippocampus at encoding is related to later autonoetic reliving. Using virtual reality (VR), during the encoding of virtual scenes, we manipulated BSC by varying the level of visuomotor (feedback of participants’ right-hand movements) and perspectival congruency (first-person versus third-person perspective), while recording brain activity, and collected ANC 1 week after encoding by established questionnaire procedures. Our results show that changes in functional connectivity strength between left hippocampus and left dPMC (contralateral to hand movements) were associated with different levels of visuomotor and perspectival congruency. Moreover, this connectivity modulated the relationship of SoA during encoding with later subjective ANC during reliving. Thus, higher connectivity was associated with a stronger SoA–ANC association, while lower connectivity led to a weaker association. Critically, this modulatory effect was absent for dPMC–hippocampus connectivity in the right hemisphere (i.e., ipsilateral to hand movements). These findings support the role of dPMC, a key SoA region, in ANC, linking subjective bodily experience during encoding to the subsequent subjective re-experiencing of past events.

## 1 Introduction

Autonoetic consciousness (ANC) is a core feature of episodic memory, enabling individuals to mentally relive events from the past (Tulving, 1985; Wheeler et al., 1997) by retrieving sensory, emotional, and personal details linked to the specific time and place in which the event occurred. Thus, ANC includes both a sense of self in the past (during the encoding of events) and a sense of self in the present (during the reliving of that past event), linking memory and the sense of self. Despite the importance of ANC in memory, our identity, and sense of self (i.e., Gardiner et al., 2001; Irish et al., 2011; Markowitsch & Staniloiu, 2011; Piolino et al., 2006; Tulving, 1985; Wheeler et al., 1997), current understanding of the neural underpinning of ANC remains limited, likely due to the difficulty in controlling the events that are encoded in one’s life (outside the laboratory) and the information that is retrieved during recollection as well as the inherent complexity related to the quantification of subjective data.

Concerning the neural underpinning of ANC, clinical observations have reported deficits involving ANC subsequent to damage to the hippocampus and/or the prefrontal cortex (Gomez et al., 2012; Illman et al., 2011; Klein & Nichols, 2012; Piolino et al., 2003). ANC deficits have also been reported in patients with a frontal variant of frontotemporal dementia and patients with Alzheimer’s disease (Piolino et al., 2006). However, most of these studies reported ANC deficits as well as deficits in episodic and autobiographical memory, making it difficult to isolate the neural underpinning specific to ANC versus broader episodic memory deficits. Moreover, Klein and colleagues proposed that ANC is associated with the sense of self as related to bodily perception and sensorimotor processing (Klein et al., 2004, 2011), often referred to as bodily self-consciousness (BSC) (Blanke et al., 2015; De Vignemont, 2011; Ehrsson, 2007; Park and Blanke, 2019; Tsakiris, 2010). This minimal-bodily self or BSC includes subjective experiences such as the sense of agency (SoA; the feeling that one is in control of its body and its actions) and the sense of body ownership (SoO; the feeling that one’s body belongs to oneself). Klein and colleagues proposed, but did not test experimentally, that SoA and SoO are important in ANC (Klein et al., 2004, 2011). More generally, and despite the importance of clinical data in neuroscience, these clinical data on ANC and the relation between ANC and BSC were obtained in neurological and/or psychiatric patients and may thus not extend to the brain mechanisms of ANC in healthy individuals.

In the last two decades, the use of virtual reality (VR) has made it possible to expose participants to different visuomotor conflicts leading to changes in SoA (i.e., Fourneret & Jeannerod, 1998; Kannape & Blanke, 2013; Padilla-Castañeda et al., 2014), although some studies have also reported changes in SoO (Salomon et al., 2016). By contrast, visuotactile mismatch has been reported to alter SoO (Ehrsson, 2007; Lenggenhager et al., 2007) while not altering SoA (Girondini et al., 2025), although most of the studies to date did not gauge SoO and SoA together when performing these experiments. Moreover, perspectives differing from the habitual first-person and embodied view have also been shown to decrease the SoA, as well as SoO (Debarba et al., 2017; Kokkinara et al., 2016; Ruby & Decety, 2001; Scattolin et al., 2022) likely related to the interference with BSC mechanism related to peripersonal space (i.e. Blanke et al., 2015; Noel et al., 2018). Thus, VR enables the disruption of multisensory, sensorimotor, and perspectival mechanisms, thereby inducing changes in BSC typically including SoA and/or SoO. More recently, researchers have used such manipulations to better understand the impact of BSC on episodic memory as well as ANC in healthy participants. These studies provided behavioral evidence for the role of BSC in episodic memory by showing that a reduced SoA and SoO during encoding leads to a reduction of memory accuracy (Bergouignan et al., 2014; Bréchet et al., 2019; Gauthier et al., 2020; Iriye & Ehrsson, 2022; Meyer, Gauthier, Stampacchia, et al., 2024) and a reduction of selected subjective aspects of re-experiencing of the event, such as the emotional intensity or vividness of reliving (Iriye & Ehrsson, 2022).

From these latter studies, only few investigated the neural underpinnings that link BSC and episodic memory, converging on the key role of the hippocampus in linking BSC and episodic memory (Bergouignan et al., 2014; Iriye et al., 2024; Meyer, Gauthier, Stampacchia, et al., 2024). The hippocampus is known to play a key role in episodic memory, and to be critical in the hippocampal–neocortical axis, where its activation mediates the reactivation of cortical areas previously involved during the encoding of an event (Lavenex & Amaral, 2000; McCormick et al., 2015; Moscovitch et al., 2016; Nadel et al., 2000; Sekeres et al., 2018). However, it is currently unknown whether the hippocampal–necortical axis is relevant for the coupling of BSC and ANC. One study manipulating SoO reported that activity in the angular gyrus, that has been linked to BSC (Blanke et al., 2002; Ionta et al., 2011) and episodic memory (Bréchet et al., 2018; Humphreys et al., 2021), predicts events encoded with different levels of the SoO (Iriye & St. Jacques, 2025; Iriye et al., 2024). Recently, our group reported a positive coupling during an episodic memory task between the reinstatement of the left hippocampus and the left dorsal premotor cortex (dPMC; known to be involved in the SoA and SoO), which was further dependent on the level of BSC at encoding (Meyer, Gauthier, Stampacchia, et al., 2024). Both studies describe the combined involvement of the hippocampus and BSC-related cortical areas related to episodic memory performance (dPMC, angular gyrus), but neither of them has reported on their involvement in the explicit subjective reliving of past events (ANC).

Recently, we reported that the level of SoA at encoding predicted the level of ANC (acquired 1 week after the encoding of scenes), but only when the event was encoded with normal-preserved BSC (Meyer, Gauthier, Potheegadoo, et al., 2024). Using an ANC questionnaire originally tailored for autobiographical event to measure ANC for scenes encoded in VR under different levels of BSC (SoA, SoO) (Meyer, Gauthier, Stampacchia, et al., 2024; Meyer, Gauthier, Potheegadoo, et al., 2024), we here extend our BSC–ANC investigation to the neural mechanism supporting this association by investigating whether encoding-related functional connectivity between the left hippocampus and left dPMC (as reported in (Meyer, Gauthier, Stampacchia, et al., 2024) also mediates BSC–ANC coupling.

## 2 Methods

All participants provided written informed consent and were financially compensated for their participation. The main imaging study included 29 participants (11 males, 3 gender-nonconforming, mean age: 24 ± 3.4 years). However, five participants were excluded due to technical issues or excessive motion during the scanning session. In addition, we used data from two other similar experiments done without imaging acquisition (49 participants; 17 males, mean age: 25 ± 4 years) for behavioral analysis. All participants were right-handed, as determined by the Flinders Handedness Survey (FLANDERS, Nicholls et al., 2013), and had no history of neurological or psychiatric disorders or drug consumption in the 48 hours prior to the experiment. The study was approved by the local ethical committee (Cantonal Ethical Committee of Geneva: 2015-00092, and Vaud and Valais: 2016-02541) and respected the declaration of Helsinki (2013).

### 2.1 Material and technical setup

The material and technical setup have been reported in our previous study (Meyer, Gauthier, Potheegadoo, et al., 2024). In this section, we summarize only the relevant aspects of the setup for the present study. For more detail refer to Meyer, Gauthier, Potheegadoo, et al. (2024).

The experiments consisted of three sessions: An encoding session, a recognition task 1 hour after the encoding (results have been reported in Meyer, Gauthier, Stampacchia, et al., 2024), and a questionnaire to quantify autonoetic consciousness (ANC). For the scope of this paper, we will focus on the ANC assessment and its link with the functional connectivity measured in the main imaging study.

Participants were lying in an MR scanner (main MRI study) or in a Mock scanner (for behavioral studies) while observing different scenes in VR and holding custom response devices in their hands to simultaneously record participants’ answers and track participants arm movements.

### 2.2 Encoding session

**Fig. 1.**
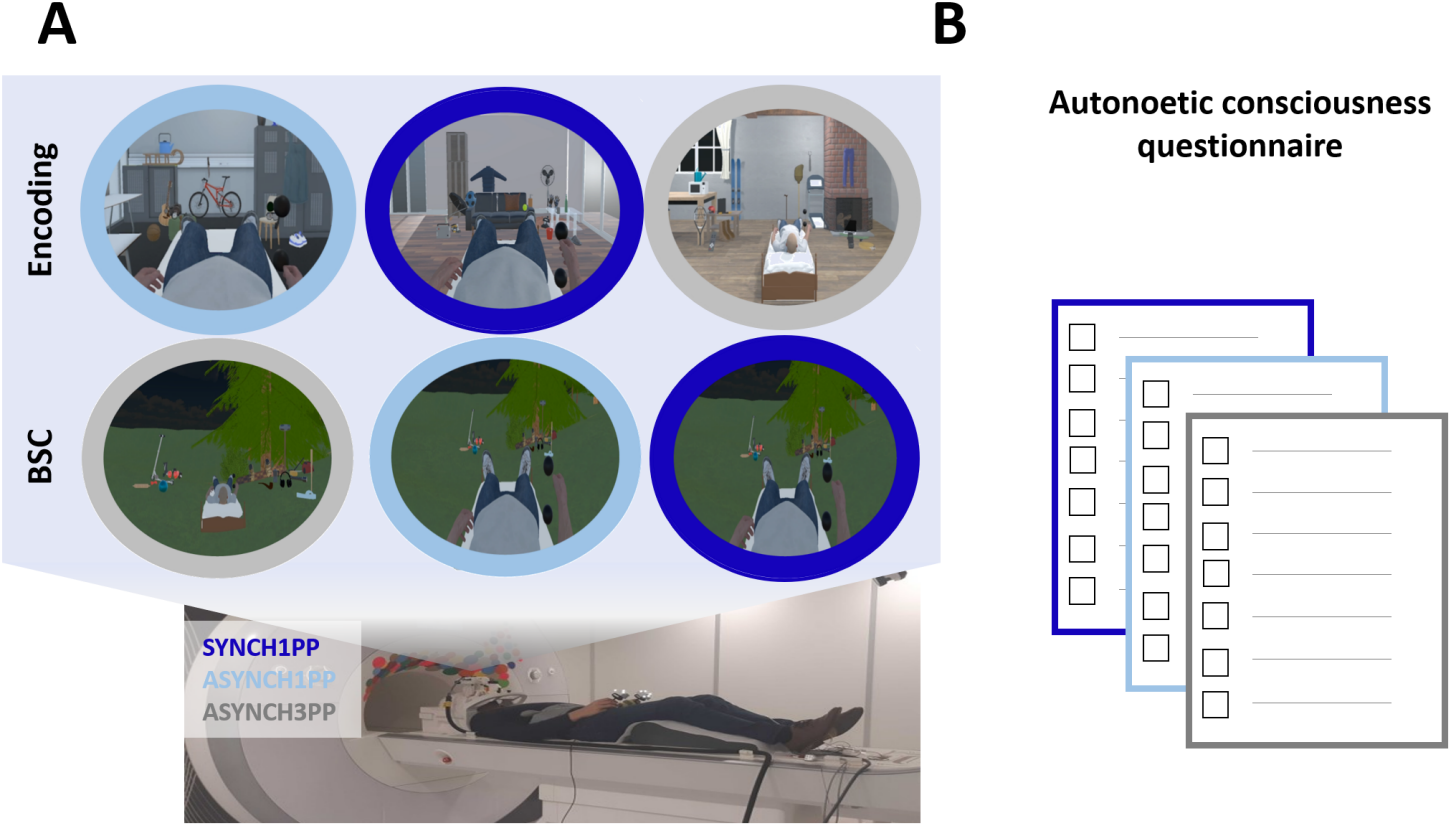
Experimental design. (A) Encoding and BSC assessment. Participants were in an MR scanner (main MRI study) or a Mock scanner (for the behavioral study) while observing different scenes in virtual reality. During encoding, participants moved their right arm while observing an avatar performing the same movement. Participant encoded one scene under synchronous first-person perspective (SYNCH1PP, preserved BSC, dark blue), one scene under asynchronous first-person perspective (ASYNCH1PP, light BSC manipulation, light blue), and one scene under asynchronous third-person perspective (ASYNCH3PP, strong BSC manipulation, grey). BSC was assessed immediately after encoding in a different fourth scene, where participants rated their sense of agency (SoA), body ownership (SoO), and control questions, for each condition. (B) Autonoetic consciousness assessment. One week later, we evaluated autonoetic consciousness (ANC) of participants using a series of questions derived from standardized questionnaires (“memory characteristic questionnaire”; MCQ, Johnson et al., 1988) and the “episodic autobiographical memory interview”; EAMI part B, Irish et al., 2008, 2011). See Table 1 for the detail of each item. The figure is adapted from Meyer, Gauthier, Potheegadoo, et al. (2024).

During encoding, participants moved their right arm between two virtual spheres while observing an avatar in three indoor virtual scenes, each presented for 30 seconds. This was repeated four times; thus, each scene was observed for 2 minutes. Each scene varied in visuomotor and perspectival congruency (Fig. 1A): thus, each participant encoded one scene under synchronous first-person perspective (SYNCH1PP, preserved BSC), one scene under asynchronous first-person perspective (ASYNCH1PP, light BSC manipulation), and one scene under asynchronous third-person perspective (ASYNCH3PP, strong BSC manipulation). Scene-condition associations were pseudorandomized across participants.

BSC was assessed immediately after encoding in a fourth scene (for more details see Meyer, Gauthier, Potheegadoo, et al., 2024): participants were observing the same avatar and had to perform the same movement as during the encoding session. They were asked to perform the movement under one condition for 30 seconds, after which they had to answer to one question about SoA (“I felt that I was controlling the virtual body”), one for SoO (“I felt that the virtual body was mine”), and two control items, unrelated to SoA and SoO (“I felt that I had more than three bodies”; “I felt that the trees were my body”). This was repeated for each condition, twice, leading to two ratings for each measure of BSC (SoA, SoO, and control questions). The questions were presented with a prerecorded voice while a grey screen with a horizontal slider was displayed in VR. Participants had to press a left button to stop the slider and press a right button to confirm their answer. The slider was moving between a scale of 0 (“Totally disagree”) and 1 (“Totally agree”).

### 2.3 Autonoetic consciousness

One week later, we evaluated autonoetic consciousness (ANC) of participants via phone. We asked participants to remember the encoding session of each scene, and cued them with the name of the scene (“living room,” “cabin,” and “changing room”) prior to asking them a series of questions derived from standardized questionnaires (Fig. 1B, “memory characteristic questionnaire”; MCQ, Johnson et al., 1988) and the “episodic autobiographical memory interview”; EAMI part B (Irish et al., 2008, 2011; the French version was derived from Potheegadoo et al., 2012, 2013). See Table 1 for the detail of each item.

**Table 1.** Factor analysis of ANC questionnaire.

	Items	Scale	Weight ANC-1	Weight ANC-2	Weight ANC-3
E1	My memory for this event is	1-7 Dim/Clear	0.8	-0.24	
E2	My memory for this event involves visual details	1-7 Little/ A lot	0.69	-0.46	
E3	My memory for this event involves sound	1-7 Little/A lot	0.42	0.44	
E4	My memory for this event involves smell	1-7 Little/A lot	0.3	0.41	0.48
E5	My memory for this event involves touch	1-7 Little/A lot	0.32	0.38	
E6	My memory for this event involves taste	1-7 Little/A lot	0.25	0.39	0.52
E7	My memory for this event is	1-7 Sketchy/very detailed	0.86	-0.26	
E8	My memory for the location where the event takes place is	1-7 Vague/Distinct	0.75		-0.29
E9	The relative spatial arrangement of objects in my memory for the event is	1-7 Vague/Distinct	0.73	-0.3	
E10	The relative spatial arrangement of people in my memory for the event is	1-7 Vague/Distinct	0.21	0.9	-0.35
E11	My memory for the time when the event takes place is	1-7 Vague/Distinct	0.35	0.27	-0.38
E12	The overall tone of the memory is	Negative/Neutral/Positive	0.27		
E13	The intensity of the overall tone of the memory is	1-7 Weak/Strong	0.74		
E14	In this event I was	A spectator (0)/a participant (1)	Removed from analysis due to low MSA (MSA = 0.28)		
E15	I remember how I felt at the time when the event took place	1-7 Not at all/Definitely	0.63	0.18	-0.24
E16	I remember what I thought at the time	1-7 Not at all/Definitely	0.56	0.28	-0.39
E17	When I think about or tell this memory, I feel like I relive it as it happened	1-7 Not at all/Definitely	0.52		-0.12
E18	When I remember the event, I see myself entirely in the scene as if I was watching a movie	1-7 Not at all/Definitely	0.37		
E19	I remember the event through my own eyes as during the event	1-7 Not at all/Definitely	Removed from analysis due to low MSA (MSA = 0.36)		
E20	I remember the movements and gestures I made with my body at the time of the event	1-7 / Vague/Distinct	0.17	0.46	-0.35
EAMI1	When you recall this event how would you describe it in terms of vividness?This can apply to the richness of sights, sounds, smells, tastes, touch, and any movements you may have made.	1-7 Very vague/very vivid	0.6	-0.32	0.13
EAMI2	When you recall this event, are you viewing the scene through your « own eyes » or can you see yourself in the memory from a third-person perspective?	Own eyes (5)/Mixture (4) /Third person (3) /something different(2) /no imagery (1)	Removed from analysis due to low MSA (MSA = 0.42)		
EAIM3	When you picture this event, do you visualize it as a continuous video that plays with break, moving video clips with some breaks, one moving image or is it more like a set of snapshot with no movement, or something else?	One smooth video (7)/video clips with breaks (6)/one moving image (5)/snapshot in sequence (4)/one static snapshot (3)/Hazy image (2)/no image (1)	Removed from analysis due to low MSA (MSA = 0.37)		
EAMI4	How often would you estimate you have thought about this memory since it first occurred?	Never (1)/Rarely (2)/Occasionnaly(3)/Frequently (4)	0.37	0.32	0.2
EAMI5	How often would you estimate you have spoken about this memory since it first occurred?	Never (1)/Rarely (2)/Occasionnaly(3)/Frequently (4)	0.23	0.34	
EAMI6	When you think about this event now, do you re-experience any of the emotion you originally felt at the time?To what extent are you re-experiencing this emotion as a percentage?	0/25/50/75/100%	0.59		0.2
EAMI7	Would you say you are reliving this memory or looking back on it?	Reliving (1)/Looking back (0)	0.35		0.29
EAMI8	To what extent are you re-experiencing this memory as a percentage?	0/25/50/75/100%	0.62		0.3

### 2.4 Behavioral data analysis

Behavioral analysis was applied using R (R Core Team, 2022), R Studio (RStudio, 2022), and Python. Linear mixed models were computed in R using the package *lme4* and *lmerTest.*

### 2.5 Autonoetic consciousness score

We extracted a measure of ANC for each participant in two different ways. First, we summed the normalized ratings (to obtain a score between 0 and 1 for each item) of each participant, to obtain one ANC score per participant and condition (as in Meyer, Gauthier, Potheegadoo, et al., 2024). We will refer to this score as the condensed autonoetic consciousness score. As we used a questionnaire that was designed for autobiographical event, the condensed ANC score computed as the sum of the ratings of the 28 questions might contain unwanted noise as some questions might be irrelevant to our virtual scenarios. To reduce that noise, we applied a factor analysis to the questionnaire, using the data from the main study and the behavioral studies. We used a parallel analysis (from the package “psycho” in R, function fa.parallel from Makowski, 2018), to determine the number of factors suitable for that analysis. The analysis was performed on responses to the ANC questionnaire collected in the SYNCH1PP condition, as this condition was characterized by preserved sensorimotor and perspectival congruency and thus was not expected to show a reduced ANC score. We then apply a factor analysis on the ANC questionnaire using the minimum residual extraction method and no rotation as implemented in the fa function from the psych package. Regression-based factor scores were computed for each participant and each factor. We applied a Kaiser-Meyer-Olkin (KMO) measure of sampling adequacy (MSA) to look at the MSA value of each item. Items with MSA lower than 0.5 were removed due to poor shared variance (Kaiser & Rice, 1974). The factor analysis was then recomputed on the remaining item, and the score of participants was also recomputed and used for further analysis.

We will refer to each of these subscores as ANC- 1, -2, and -3.

### 2.6 Bodily self-consciousness

To confirm that our experimental manipulation effectively reduced BSC in the ASYNCH1PP and ASYNCH3PP conditions, we applied a linear mixed model with condition as a fixed effect and participants as a random effect to explain variations in SoA /SoO. To ensure comparability between the MRI and behavioral data, we included experiment type (MRI vs. behavioral) as an additional fixed factor in the model. We applied the same model using the mean of the control ratings as dependent variable to ensure that the effect captured was not due to experimental bias.

### 2.7 Autonoetic consciousness and bodily self-consciousness

We previously reported that the interaction between SoA and the conditions could explain ANC score (reported in Meyer, Gauthier, Potheegadoo, et al., 2024) using the following model:

ANC ~ SoA * Conditions+random effect of participants. To verify that the bodily subjective experience (SoA and SoO) modulated the subscore of ANC similarly to the condensed score of ANC (reported in Meyer, Gauthier, Potheegadoo, et al., 2024), we applied the same linear model reported in Meyer, Gauthier, Potheegadoo, et al. (2024), replacing the ANC condensed score by each ANC subscore. We also added the experiment as a covariate (2 factors: MRI experiment and behavioral experiment) to make sure that the data from the MRI experiment were similar to the data from the behavioral experiment. Thus, the model explained each ANC subscore (dependent variable) with the conditions and interaction with the bodily subjective experience (SoA/SoO) score:

$$\begin{array}{l} \text{ANC subscore }\sim \text{Conditions * SoA}\;/\;\text{SoO }\\ \;\;+\text{Experiment}+\text{random effect of participants}. \end{array}$$

Finally, as a control we applied the same model for the control question (the mean of the two control questions). We used the SYNCH1PP condition as the reference condition to evaluate the model. Since each model was applied three times (using SoA, SoO, and Control as interaction term), we applied a Bonferroni correction so that the significance level would be p = 0.05/3 = 0.017 for each model.

### 2.8 MRI analysis

The MR images acquisition parameters and preprocessing steps are reported in Meyer, Gauthier, Stampacchia, et al. (2024). We used the Conn toolbox (release 20.b, www.nitrc.org/projects/conn) and SPM12 (release 12.7771) to denoise the functional data using a standard denoising pipeline including the regression of potential confounding effects characterized by white matter time series, CSF time series, motion regressors, and linear trends within each functional run, followed by bandpass frequency filtering of the BOLD time series between 0.008 Hz and 0.09 Hz.

#### 2.8.1 General psychophysiological interaction

To investigate the functional changes related to the experimental conditions at encoding, we applied a psychophysiological interaction analysis. Seed regions included left dorsal premotor cortex (dPMC), and left hippocampus, identified in previous studies using the same encoding paradigm (Meyer, Gauthier, Stampacchia, et al., 2024). Additionally, these two seeds were reversed on the right hemisphere to investigate whether the effect was specific to the hemisphere which was contralateral to the upper limb movements (the left hemisphere) or bilaterally equivalent. In the text, we refer to these reverse regions as the right hippocampus and right dPMC. A generalized psychophysiological interaction model (gPPI) was defined separately for each pair of seed and target areas, with seed BOLD signals as physiological factors, boxcar signals characterizing each individual task condition convolved with an SPM canonical hemodynamic response function as psychological factors, and the product of the two as psychophysiological interaction terms. Functional connectivity changes across conditions were characterized by the multivariate regression coefficient of the psychophysiological interaction terms in each model.

#### 2.8.2 Premotor–hippocampal functional connectivity

Previous data in the same participants (Meyer, Gauthier, Stampacchia, et al., 2024) linked the SoA to episodic memory and to the reinstatement (recorded 1 hour after the encoding session) of left dPMC activation (MNI coordinates: -18, -25, 62) and left hippocampus activation (MNI coordinates: -25, -22, -15). Based on these data, we here investigated whether changes in functional connectivity between left dPMC and left hippocampus during the encoding of scene were sensitive to the different experimental conditions (BSC). For each participant and each condition, we extracted the functional connectivity strength between the nodes and applied a linear mixed model to evaluate whether the functional connectivity strength was different between conditions.

$$\begin{array}{l} Functional\;connectivity_{left\;hippocampus-left\;dPMC}\;\\ \;\;\sim \;Conditions+random\;effect\;of\;participant \end{array}$$

$$\begin{array}{l} Functional\;connectivity_{left\;dPMC-left\;hippocampus}\\ \;\;\sim \;Conditions+random\;effect\;of\;participant \end{array}$$

Since we applied two tests with the same hypothesis, we corrected for multiple comparisons using the Benjamini–Hochberg correction.

The same analysis was applied to the same regions in the right hemisphere (both seed regions were mirrored to the right hemisphere, using *ImCalc* in SPM12). Note that with the gPPI analysis, the connectivity matrix is not symmetric, meaning that the functional connectivity of seed 1 to seed 2 is not the same as the functional connectivity of seed 2 to seed 1. Our model always evaluated both functional connectivity strengths and we reported the results for both directions in the text. However, as the TR used for this study is 1.5 seconds, this difference in connectivity strength is more mathematical than biological, so we will not interpret the difference in directionality in our results.

#### 2.8.3 Autonoetic consciousness and encoding functional connectivity

As previously reported in Meyer, Gauthier, Potheegadoo, et al. (2024), there was a significant interaction between SoA and the experimental conditions to explain ANC scores (ANC ~ SoA * Conditions + random effect of participants). We found that behaviorally, ANC could be explained by SoA only under preserved visuomotor and perspectival congruency. Here, since we expected that the functional connectivity between the left hippocampus and dorsal premotor cortex differed between conditions, we performed linear mixed models to investigate whether the functional connectivity between left dPMC and left hippocampus at encoding and its interaction with bodily subjective experience SoA/SoO could explain ANC ratings of the participants, evaluated a week later. We first applied the model to explain the condensed ANC score. Because we do not have a priori hypothesis toward a specific directionality (hippocampus to dPMC or dPMC to hippocampus), we applied the model for both.

$$\begin{array}{l} ANC_{Condensedscore}\;\\ \sim \;\left(Functional\;connectivity_{left\;hippocampus\;-left\;dPMC}\right)\;\\ \;\;\;*SoA\;/\;SoO+random\;effect\;of\;participant \end{array}$$

$$\begin{array}{l} ANC_{Condensedscore}\;\\ \sim \;\left(Functional\;connectivity_{left\;dPMC-left\;hippocampus}\right)\;\;\\ \;\;\;*SoA\;/\;SoO+random\;effect\;of\;participant \end{array}$$

Then, we applied the same model to explain the different ANC subscores obtained from the factor analysis with the same variable.

$$\begin{array}{l} ANC_{Subscore1}\;\\ \sim \;\left(Functional\;connectivity_{left\;hippocampus-left\;dPMC}\right)\\ \;\;\;\;*\;SoA\;/\;SoO+random\;effect\;of\;participant \end{array}$$

$$\begin{array}{l} ANC_{Subscore1}\;\\ \sim \;\left(Functional\;connectivity_{left\;dPMC-left\;hippocampus}\right)\;\\ \;\;\;*SoA\;/\;SoO+random\;effect\;of\;participant \end{array}$$

$$\begin{array}{l} ANC_{Subscore2}\\ \;\sim \left(Functional\;connectivity_{left\;hippocampus-left\;dPMC}\right)\;\;\\ \;\;\;*SoA\;/\;SoO+random\;effect\;of\;participant \end{array}$$

$$\begin{array}{l} ANC_{Subscore2}\;\\ \sim \;\left(Functional\;connectivity_{left\;dPMC-left\;hippocampus}\right)\\ \;\;\;\;*\;SoA\;/\;SoO+\;random\;effect\;of\;participant \end{array}$$

$$\begin{array}{l} ANC_{Subscore3}\;\\ \sim \;\left(Functional\;connectivity_{left\;hippocampus-left\;dPMC}\right)\\ \;\;\;*\;SoA\;/\;SoO+random\;effect\;of\;participant \end{array}$$

$$\begin{array}{l} ANC_{Subscore3}\\ \sim \;\left(Functional\;connectivity_{left\;dPMC-left\;hippocampus}\right)\;\\ \;\;\;\;*\;SoA\;/\;SoO+random\;effect\;of\;participant \end{array}$$

For each subscore, since we applied four tests with the same hypothesis (using SoA/SoO and both directionality of functional connectivity), we corrected for multiple comparisons using the Benjamini–Hochberg correction.

We conducted sensitivity analyses for our two main models, using α = 0.05. First, for the model comparing functional connectivity between the left hippocampus and the left dorsal premotor cortex across conditions (*Functional connectivity_left hippocampus-left↔dPMC_ ~ Conditions* *+* *random effect of participant)*, we observed standardized regression coefficients (β) of 0.52 for the comparison between the SYNCH1PP and ASYNCH3PP conditions, and 0.35 between SYNCH1PP and ASYNCH1PP, indicating moderate to large effects. Second, for the linear mixed-effects model predicting ANC-2 from the interaction between sense of agency (SoA) and functional connectivity (ANC_Subscore2_ ~ (Functional connectivity_left hippocampus-left↔dPMC)_ * SoA/SoO *+* *random effect of participant)*, we observed a standardized regression coefficient of 0.27, reflecting a small to moderate effect.

These results confirm that our study was appropriately powered to detect small to moderate effects within the range observed in the literature.

## 3 Results

### 3.1 Sense of agency and sense of body ownership are reduced following visuomotor and perspectival manipulation

We first verified that our manipulation led to a significant reduction of SoA and SoO. We found that SoA was significantly reduced in the ASYNCH1PP and ASYNCH3PP conditions when compared with the SYNCH1PP condition (ASYNCH3PP: estimate = -0.08, t = -4.28, p < 0.0001, ASYNCH1PP: estimate = -0.056, t = -2.87, p = 0.005). SoO was significantly reduced in ASYNCH3PP when compared with SYNCH1PP (estimate = -0.12, t = -4.8, p < 0.0001). In both cases (SoA and SoO), there was no significant effect of experiment (behavioral versus MRI experiment). Importantly, the control questions had low ratings and were not significantly different between conditions, suggesting that the effect was not due to suggestibility (ASYNCH1PP: estimate = 0.008, t = 0.73, p = 0.46, ASYNCH3PP: estimate = -0.018, t = -1.76, p = 0.08, see supplementary Table S1 for more details).

### 3.2 Functional connectivity between left hippocampus and left dorsal premotor cortex during encoding is higher under visuomotor and perspectival congruency

**Fig. 2.**
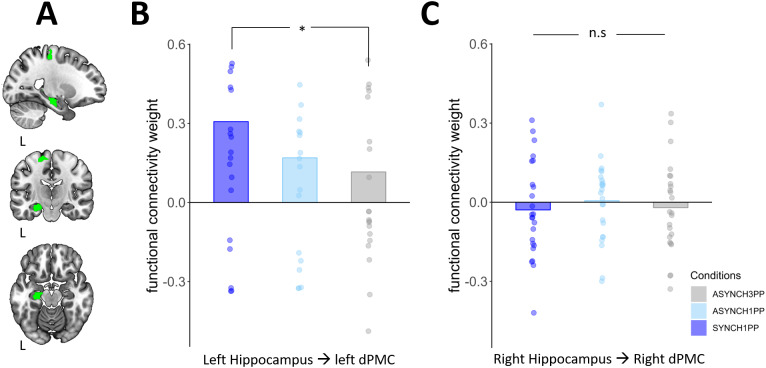
Functional connectivity strength between hippocampus and dorsal premotor cortex. (A) Region of interests. Left dPMC and hippocampus region of interest used in this study. The regions are displayed on a standard MNI template using MriCroGL, left hemisphere is indicated by the letter “L”. (B) Functional connectivity strength in the left hemisphere. Functional connectivity strength is higher for scene encoded under visuomotor and perspectival congruency (SYNCH1PP, dark blue) compared to scene encoded under strong visuomotor and perspectival mismatch (ASYNCH3PP, grey), N = 24, * indicates significance level with p-value <0.05. (C) Functional connectivity strength in the right hemisphere. There was no significant difference between conditions for the functional connectivity in the right hemisphere. N = 24. dPMC = dorsal premotor cortex.

We aimed to investigate whether the functional connectivity between two regions of interest (left hippocampus and left dPMC), as shown previously to be involved in the association between BSC during encoding and recognition memory after a 1-hour delay (Meyer, Gauthier, Stampacchia, et al., 2024), was also mediating BSC during encoding and ANC after a delay of 1 week, depending on visuomotor and perspective congruency. Additionally, we examined whether this functional connectivity could predict autonoetic consciousness measured 1 week later. We found that the functional connectivity of the left hippocampus to the left dPMC during encoding significantly differed between the SYNCH1PP and the ASYNCH3PP conditions (Fig. 2A, left hippocampus to left dPMC: estimate = -0.099, t = -2.473, p = 0.017) with a similar trend in the other direction (i.e. from left dPMC to left hippocampus, estimate = -0.19, t = -1.78, p = 0.08). The same comparison between SYNCH1PP and ASYNCH1PP also tended toward significance (left hippocampus to left dPMC: estimate = -0.068, t = -1.68, p = 0.099), with no significant difference in the other direction (left dPMC to left hippocampus, estimate = -0.13, t = -1.28, p = 0.2). These results show that the functional connectivity between the left dPMC and left hippocampus during encoding is stronger in SYNCH1PP than in ASYNCH3PP.

As the participants moved their right upper limb during encoding (associated with activation of left dPMC (Meyer, Gauthier, Stampacchia, et al., 2024), we did not expect similar connectivity changes between the right dPMC and right hippocampus (i.e., ipsilateral with respect to the participants’ right hand during the visuomotor manipulation during encoding). Indeed, there were no significant functional connectivity differences between the right dPMC and right hippocampus between conditions (Fig. 2B, SYNCH1PP compared with ASYNCH1PP, right hippocampus to right dPMC: estimate = 0.035, t = 0.854, p = 0.398, right dPMC to right hippocampus: estimate = 0.12, t = 1.201, p = 0.236, SYNCH1PP compared with ASYNCH3PP, right hippocampus to right dPMC: estimate = 0.008, t = 0.2, p = 0.836, right dPMC to right hippocampus: estimate = 0.036, t = 0.335, p = 0.739).

Further post hoc analysis revealed that the functional connectivity between the left hippocampus and left dPMC across conditions was significantly higher in the left hemisphere than in the right hemisphere (hippocampus to dPMC, left versus right hemisphere: estimate = -0.09, t = -3.6, p = 0.00046, dPMC to hippocampus, left versus right: estimate = -0.25,t = -3.94, p = 0.00014). One sample t-test further confirmed these results and showed that the functional connectivity of the left hemisphere was above 0 (left hippocampus to left dPMC: t = 3.46, p = 0.0009, left dPMC to left hippocampus: t = 3.77, p = 0.00033), while the right hemisphere’s functional connectivity did not differ from 0 (right hippocampus to right dPMC: t = -0.74, p = 0.46, right dPMC to right Hippocampus: t = -1.15, p = 0.25). These results show that the left hippocampus (involved in episodic memory) and left dPMC (involved in SoA), both contralateral to the right-hand movements used to modulate visuomotor congruency during encoding, are more strongly and more selectively coactivated when participants encoded the scenes with preserved visuomotor and perspectival congruency (SYNCH1PP).

### 3.3 Autonoetic consciousness (condensed score) is not explained by functional connectivity between left hippocampus and left dorsal premotor cortex

We previously reported that the coupling between the subjective ratings of ANC and SoA depended on the different experimental conditions (Meyer, Gauthier, Potheegadoo, et al., 2024): higher SoA was associated with higher ANC. Accordingly, we here investigated whether the functional connectivity between the left hippocampus and left dPMC at encoding and the SoA rating strength could explain the ANC ratings for the encoded scenes 1 week later. Applying the model to explain the ANC score by the left hippocampus–dPMC functional connectivity and the SoA ratings, however, revealed no significant interaction (left hippocampus to left dPMC interacting with SoA: estimate = 0.33, t = 0.93, p = 0.35, left dPMC to left hippocampus interacting with SoA: estimate = -0.11, t = -0.67, p = 0.5). Similarly, no interaction was found when we used SoO in the model (left hippocampus to left dPMC interacting with SoO: estimate = 0.94, t = 0.4, p = 0.93, left dPMC to left hippocampus interacting with SoO: estimate = 0.5, t = 0.53, p = 0.76).

### 3.4 Information reduction of the autonoetic consciousness score

We note, however, that the ANC score gives the same weight to each of the different questionnaire items. Yet, since the ANC questionnaire was developed for autobiographical events, it is possible that some questions are less or not relevant in our experiment (with laboratory-based encoding of simulated life-like events in VR) and may, therefore, alter the information captured by the total questionnaire due to the low scores given by the participants. To investigate this further, we performed the same functional connectivity analysis for each of the ANC questionnaire subfactors derived by factor analysis. This allowed us to give different weights for certain items based on their relevance to explain the variability in ANC scores between subjects. We identified that three factors were necessary to optimize the information carried out by the ANC questionnaire (supplementary Fig. S1). Table 1 depicts the weight attributed for each item of the questionnaire (after applying the factor analysis with three factors; see methods for details). We removed four items that had a low MSA score (< 0.5), prior to the final factor analysis (see Table 1). After completion of the factor analysis, the global MSA was of 0.78, indicating a good level of adequacy for factor analysis (Kaiser & Rice, 1974). The first factor (ANC-1) explained 25% of the questionnaire variance and attributed higher weight to items related to the visual memory, and vividness, whereas the second factor (ANC-2) explained 7% of the variance of the original questionnaire and attributed high weight for non-visual sensory item (such as memory for tactile and auditory stimuli) as well as for verbal recall (how much participants reported talking about the event). Finally, the third factor (ANC-3) explained 6% of the original questionnaire and attributed high weight for questions related to olfactory and gustatory items, which had low between-subject variability and low scores between participants. Therefore, ANC-3 likely captures noise rather than a meaningful construct. Similarly to the significant interaction obtained between SoA and visuomotor–perspectival congruency to explain the condensed ANC score reported in our previous study (Meyer, Gauthier, Potheegadoo, et al., 2024), we found a significant interaction between the SoA and the experimental manipulation of visuomotor and perspectival congruency to explain the first ANC subscore (ANC-1) obtained from the factor analysis (SYNCH1PP compared with ASYNCH3PP: estimate = -1.16, t = -3, p = 0.004, for the rest of the model’s result see supplementary Table S2). Importantly, post hoc analyses revealed that SoA was significantly associated with ANC only in the SYNCH1PP condition (estimate = 1.00, t = 2.08, p = 0.041), while no significant coupling was found in the ASYNCH1PP (estimate = -0.04, t = -0.18, p = 0.86) or ASYNCH3PP conditions (estimate = -0.14, t = -0.67, p = 0.50). The two other factors did not show a significant interaction (See Supplementary Tables S2-S4). When applying the same model using SoO as interaction term to predict the different factor, there was again no significant interaction (Supplementary Tables S5-S7). Together these results suggest that the ANC reduction based on the factor analysis preserved the previously reported behavioral SoA–ANC effect.

### 3.5 ANC subscore is explained by functional connectivity between left hippocampus and left dorsal premotor cortex

**Fig. 3.**
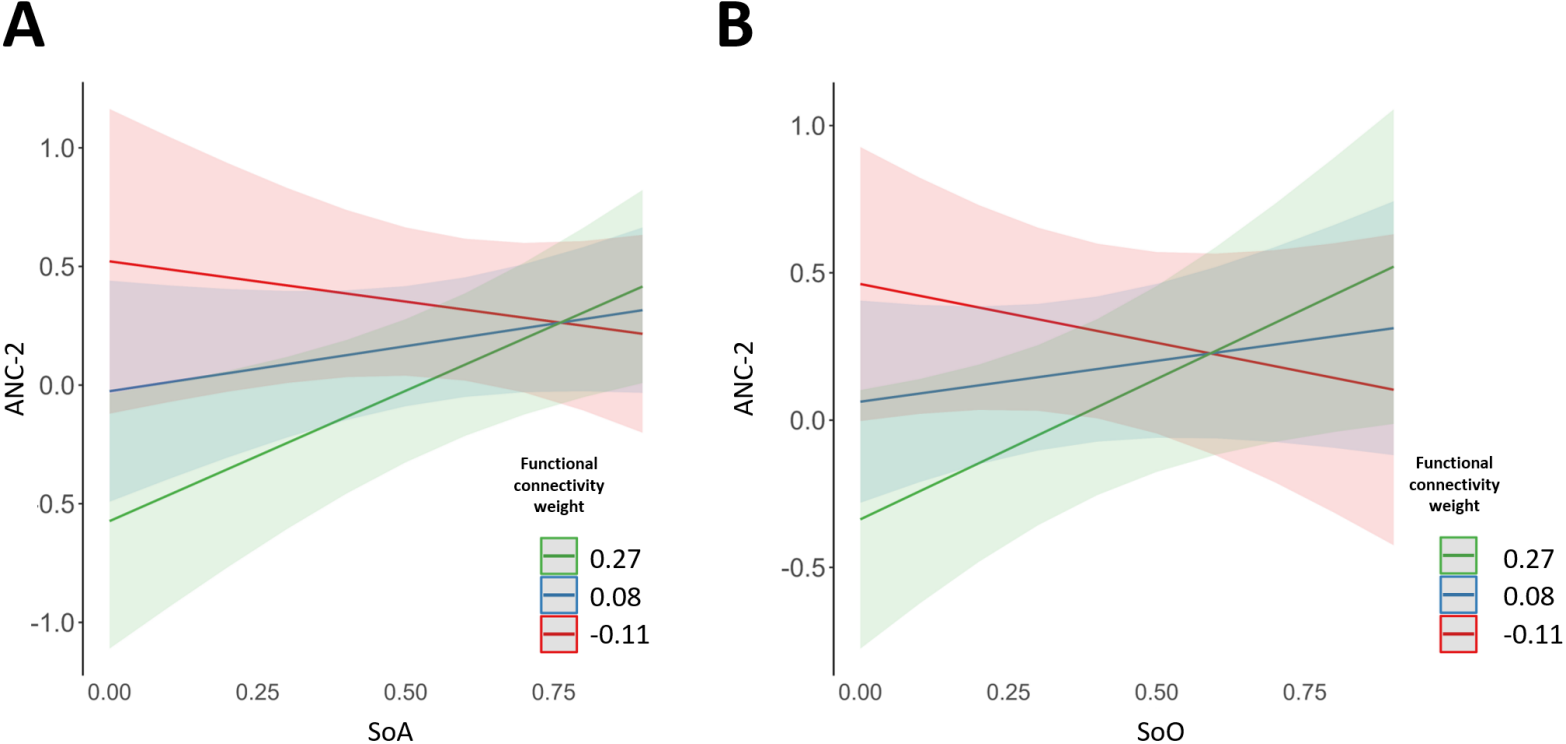
ANC subfactor 2 (ANC-2) is explained by the interaction of the encoding functional connectivity between hippocampus and dPMC and bodily subjective experience. (A) premotor–hippocampal functional connectivity and SoA. There is a significant interaction between SoA and the functional connectivity of the left hippocampus and dPMC to explain ANC-2. When the functional connectivity at encoding is high (green line), there is a positive relationship between SoA and ANC, whereas this relationship is lost for lower functional connectivity values (blue and red lines). (B) Premotor–hippocampal functional connectivity and SoO. We found a similar interaction when using SoO as interaction term with ANC-2. N = 24. dPMC = dorsal premotor cortex, ANC = autonoetic consciousness.

When we applied a linear mixed model to explain the three ANC subscores by the functional connectivity between the left hippocampus and the left dPMC, we found a significant interaction of the functional connectivity between the left hippocampus and left dPMC with SoA to explain ANC-2 (left hippocampus to left dPMC: = 3.78, t = 2.72, p = 0.009, p_corrected_ = 0.034). ANC-2 is a subfactor with high weight on sensory items (auditory and tactile) as well as items related to memory of bodily movement and verbal recall and low weight related to visual items (see Table 1). Figure 3 showed that participants with high functional connectivity between left hippocampus and left dPMC had a positive correlation between SoA and ANC-2 ratings, whereas for lower functional connectivity, there was no relationship between SoA and ANC-2. Other ANC-subscores did not reveal significant results (Supplementary Tables S8-S13). Concerning SoO, we found similar effect for ANC-2 (significant interaction between SoO and premotor–hippocampal functional connectivity with ANC-2; left hippocampus to left dPMC: estimate = 3.56, t = 2.4, p = 0.02, p_corrected_ = 0.038; Supplementary Tables S14-S19). To ensure the relationship was specific to SoA and SoO, we applied the same model to explain ANC-2 by the premotor–hippocampal connectivity and its interaction with the subjective control ratings (which were used to measure potential experimental bias and, therefore, are not expected to show significant effect of interaction). We found no significant interaction (left hippocampus to left dPMC, estimate = 4.76, t = 1.84, p = 0.07). Finally, to test the laterality of the effect mentioned above, we applied the same model using the functional connectivity of the hippocampus and dPMC in the right hemisphere (hemisphere ipsilateral with respect to the right hand modulating BSC) and found no significant relationship between SoA/SoO and ANC (same models with the functional connectivity of the right dPMC and right hippocampus; all p > 0.25; Supplementary Tables S20-S31). These results show that the functional connectivity between the left hippocampus and the left dPMC during encoding is an important parameter that modulates the relationship between subjective bodily experience (SoA and SoO) during the encoding of events and later reliving (ANC) of these events. Thus we found that when the left hippocampus and the left dPMC are coactivated during the encoding of scenes, a stronger subjective bodily experience is associated with a stronger subjective reliving of the scenes 1 week later (ANC strength). This ANC–SoA/SoO relationship was absent for weaker premotor–hippocampal coactivation and only absent in the right ipsilateral hemisphere.

## 4 Discussion

Extending previous work that linked BSC with episodic memory in a 1 hour-delay recognition task, associated with premotor–hippocampal (dPMC-hippocampus) reinstatement (Meyer, Gauthier, Stampacchia, et al., 2024), we here investigated whether functional connectivity between these two regions also associated BSC during encoding to ANC ratings, as recorded 1 week after the encoding session. We experimentally modulated the level of SoA and SoO during the encoding of virtual scenes using VR, by exposing participants to different gradients of visuomotor and perspectival mismatch, induced by repeated right-hand movements during encoding and collected ANC ratings 1 week after the encoding session. We report (1) that changes in functional connectivity strength between the left hippocampus and left dPMC depend on the level of visuomotor and perspectival congruency. This difference (2) was only found in the left hemisphere, contralateral to our experimental sensorimotor manipulation based on repeated right-hand movements that we used to modulate SoA/SoO and was absent in the right ipsilateral hemisphere. We further report that (3) these changes in left hippocampus-dPMC functional connectivity strength were critical to modulate the coupling between the subjective bodily experience during encoding and the subjective reliving (ANC) 1 week later, by showing that the stronger the functional connectivity between the left hippocampus and the left dPMC, the stronger the BSC–ANC coupling. Collectively, these data link premotor–hippocampal functional coupling and SoA/SoO levels during the encoding of events with the subjective re-experiencing of these past events (ANC).

### 4.1 Functional connectivity strength between hippocampus and dPMC is related to the experimental conditions

Previous work has reported a stronger functional connectivity between the hippocampus and parahippocampus *after* the encoding of scenes encoded under a naturalistic body view (1PP) compared with scenes encoded with no body view (Gauthier et al., 2020). The present results extend these findings, showing stronger functional connectivity between the left hippocampus and left dPMC *during* the encoding of scenes encoded under visuomotor and perspectival congruency (i.e., congruent sensorimotor stimulation and related state of preserved BSC). Numerous studies have highlighted the central role of the hippocampus for the formation of the memory trace (Bartsch et al., 2011; Ben-Yakov & Dudai, 2011; Dudai & Eisenberg, 2004; Josselyn et al., 2015; Squire, 1992; Squire et al., 2015; Tanaka & McHugh, 2018). Furthermore, the hippocampus is considered at the top of the hierarchy of the medial temporal lobe, integrating signals from entorhinal cortex and receiving sensory cortical input (Lavenex & Amaral, 2000; Shimamura, 2010; Tanaka & McHugh, 2018). Accordingly, we interpret the higher dPMC-hippocampus connectivity strength found under visuomotor and perspectival congruency during encoding as reflecting a stronger co-activation between the hippocampus and dPMC for conditions encoded with preserved sensorimotor integration.

Hippocampal–neocortical axis theory proposes that the hippocampus acts as a “mediator” to reactivate the different brain regions that were involved at encoding. Previously, we reported (Meyer, Gauthier, Stampacchia, et al., 2024) that hippocampal reinstatement (the reactivation of encoding-related activity during retrieval) was positively associated with dPMC reinstatement only for scenes encoded under preserved visuomotor and perspectival congruency. Here we extend this finding to functional connectivity at encoding: we show that the hippocampus is coupled with neocortical areas during encoding and that this coupling depends on the level of visuomotor and perspectival congruency at encoding. We speculate that this stronger co-activation between hippocampus and dPMC during the encoding of events may lead to a facilitation effect which could explain the stronger coupling between hippocampus–dPMC reinstatement reported in Meyer, Gauthier, Stampacchia, et al. (2024).

Critically, a similar change of functional connectivity strength, as found between left hippocampus and left dPMC, was not observed in the corresponding regions of the hemisphere ipsilateral to the hand used to modulate visuomotor congruency (i.e., the right hemisphere). As activity in dPMC contralateral to the hand movements for which SoA is tested has been frequently reported (Haggard, 2017; Seghezzi et al., 2019), our results suggest that the observed dPMC–hippocampus coupling was related to the visuomotor manipulation during encoding of the right arm of our participants (where the SYNCH1PP condition correspond to a preserved sensorimotor integration and thus preserved BSC, and ASYNCH1PP and ASYNCH3PP correspond to a light and strongly disrupted sensorimotor integration and thus, disrupted BSC). Moreover, we also observed that the left-lateralized functional connectivity between hippocampus and dPMC was positive and differed from zero across all conditions, while the corresponding values in the right hemisphere did not differ from zero (Fig. 2). These findings describe a general increase in functional connectivity that depends on the experimental conditions only for contralateral dPMC–hippocampus connectivity. As our study used only right-hand movement, future studies should further investigate the role of movements and their related activity on the contralateral hemisphere and how it is linked with ANC.

### 4.2 The coupling between subjective bodily experience (SoA/SoO) and subjective reliving (ANC) depends on functional connectivity strength between hippocampus and dPMC

ANC has recently been linked with subjective bodily experience at encoding by Iriye and Ehrsson (2022), who showed that strong versus weak body ownership was linked to greater reliving (defined as the feeling of experiencing the event again as if it were happening right now, or as if you were mentally traveling back in time to when the event occurred) immediately after encoding, as well as greater intensity and vividness 1 week later. High vividness and strong body ownership during encoding also predicted greater memory reinstatement in the hippocampus (Iriye et al., 2024). Our own previous work discovered positive SoA–ANC coupling for scenes encoded under preserved visuomotor and perspectival congruency (Meyer, Gauthier, Potheegadoo, et al., 2024). While Ehrsson and colleagues employed visuotactile stimulation to modulate the SoO (Ehrsson, 2007; Iriye & Ehrsson, 2022; Lenggenhager et al., 2007), the present study based on previous work used visuomotor stimulation targeting a main modulation of the SoA (Fourneret & Jeannerod, 1998; Kannape & Blanke, 2013; Tsakiris et al., 2010). The latter effect was confirmed in the present data by our finding from factor analysis, showing that factor ANC-1 is explained by the interaction between conditions and the SoA. Interestingly, this interaction was significant when comparing the SYNCH1PP condition (preserved BSC) with the ASYNCH3PP condition (strongly manipulated BSC), while the interaction between SYNCH1PP and ASYNCH1PP was only marginally significant (p = 0.08). However, post hoc analyses showed that only the SYNCH1PP condition significantly accounted for the positive coupling between SoA and ANC. This suggests that disrupting the visuomotor signal alone may be sufficient to weaken the SoA–ANC association. In contrast, no significant interaction was observed between the ASYNCH1PP and ASYNCH3PP conditions, indicating that changing perspective alone does not further reduce this coupling.

The present fMRI results further suggest that this BSC–ANC coupling is enabled at the neural level by the strength of functional connectivity of left dPMC–hippocampus. As shown in Figure 3, the stronger the premotor–hippocampal functional connectivity, the stronger was the coupling between our participants’ SoA and ANC. These results suggest that the association between BSC and ANC in the present study is detectable already in neural activity at encoding, and is mediated by the functional connectivity strength between a key area of ANC (the hippocampus; Bartsch et al., 2011; Moscovitch et al., 2016; Squire, 1992; Tanaka & McHugh, 2018) and a key area of BSC (dPMC; Haggard, 2017; Tsakiris et al., 2010). Therefore, the co-activation of a BSC-related cortical region with the hippocampus at encoding leads to a stronger coupling between subjective bodily experience (SoA) and subjective reliving (ANC) 1 week later. Interestingly, this relationship was also supported by an additional SoO–ANC coupling, suggesting that the functional premotor–hippocampal coupling highlighted in our study reflects a more global subjective BSC experience rather than only SoA. More work is needed to disentangle any potential differential effects of SoO versus SoA on ANC. We note that although the visuomotor manipulations we used at encoding is known to manipulate SoA (Kannape & Blanke, 2013; Padilla-Castañeda et al., 2014; Weijs et al., 2021), we also employed an additional perspective manipulation, which is known to modulate SoO (Ehrsson, 2012; Grivaz et al., 2017; Lenggenhager et al., 2007) as does visuomotor stimulation (Meyer, Gauthier, Potheegadoo, et al., 2024). Therefore, while our present manipulation may have had stronger effects on the SoA (and SoA–ANC coupling), it also affected SoO. While ANC-1 is successfully explained behaviorally by the interaction between SoA and the experimental conditions, we found that ANC-2 is explained by the interaction between SoA and the premotor–hippocampal connectivity. We speculate that this distinction may result from the fact that ANC-2 may attribute higher loadings to (non-visual) sensory ANC items (particularly those of tactile sensations) as well as bodily movements, which are more closely related to dPMC function, known to be involved in motor planning and SoA (Haggard, 2017). Therefore, participants’ responses to these items might be more closely related to, and proportional to, the strength of premotor–hippocampal functional connectivity. Importantly, the observed relationship suggests that embodied aspects of memory (especially those grounded in motor and tactile experiences) are supported by premotor–hippocampal interactions, even in the context of ANC scores collected for events encoded in VR. However, ANC-1 appears to account for a much larger portion of the questionnaire’s variance and places greater weight on the visual aspects of ANC-1. We also note that autonoetic consciousness is a long-term memory process that unfolds over days, which is why neuropsychologists typically recommend assessing ANC with events that occurred at least 1 week earlier (Conway et al., 2001; Lenormand et al., 2024; Piolino et al., 2002). Accordingly, the information captured by our questionnaire cannot be attributed solely to the encoding moment. Although SoA measured at encoding was able to partly explain such mainly visual aspects of ANC, this factor is likely also shaped by consolidation processes and interactions between the encoded material and events unfolding over the next week including various factors such as sleep, emotional state, and stress (Lenormand et al., 2024; Murre et al., 2014; Rimmele et al., 2015). We, therefore, speculate that ANC-1 reflects aspects that were consolidated after the encoding session, influenced by parameters such as visual and environmental features. As such, ANC-1 may capture a dimension of ANC that is more widespread to be explained exclusively by premotor–hippocampal connectivity measured at encoding, and more likely reflects hippocampal interactions with other brain regions, or connections established after encoding. Future studies should investigate the functional connectivity of the hippocampus with the visual cortex, and the amygdala as well as other sensory regions such as auditory cortex (not manipulated in this study) at encoding and at different stages of the consolidation process as it might be more relevant to explain ANC-1. Previous research investigating the association of BSC with episodic memory and ANC treated reliving as a single construct, often averaging questions about the vividness and emotional reliving of the experience together (e.g., Bergouignan et al., 2014; Iriye & Ehrsson, 2022). Our results provide novel insights into the multifaceted nature of re-experiencing, showing that bodily self-consciousness influences not only visually driven aspects of memory, but also extends to other sensory modalities such as tactile and motor-related experiences, further showing that the use of ANC questionnaire originally tailored for autobiographical events can also be applied to virtual scenarios.

Are the present behavioral and neural findings also of relevance for patient studies and are there situations where BSC and SoA in particular are disturbed in healthy individuals? With respect to the latter issue, the SoA and relatedly SoA–ANC coupling might be affected in healthy individuals encoding events while suffering from paresthesia of the upper extremity. Local anesthesia of isolated body parts or pain has also been shown to reduce SoA/SoO (Bullington, 2009; Svenaeus, 2015; Zeiler, 2010). Concerning neurological patients, individuals with deficits in sensorimotor integration (e.g., patients with damage to motor and premotor cortex) may exhibit reduced SoA–ANC coupling. Moreover, patients with schizophrenia are known to have impaired SoA (Corcoran & Frith, 2003) and have also reduced autobiographical memory (Berna et al., 2015; Potheegadoo et al., 2012, 2013; Riutort et al., 2003). Our SoA–ANC findings suggest that in such populations, diminished premotor–hippocampal connectivity may underlie memory and SoA–ANC deficits, due to unsuccessful integration of bodily signals at encoding. Similarly, patients with amnesia or bilateral hippocampal damage may show reduced re-experiencing of past events for the same reason. In support of this, our group reported a single-case study of a patient with bilateral hippocampal atrophy, who demonstrates better memory for events encoded under disembodied conditions. This case suggests that hippocampal atrophy selectively impairs memory for embodied experiences (Meyer, Babo-Rebelo et al., 2025), pointing to the existence of distinct encoding pathways depending on the success of sensorimotor integration. Together, our results provide evidence for a functional association between BSC and ANC brain regions during encoding, and suggest that injury to these regions may disrupt the BSC–ANC link.

Taken together, we used VR to manipulate BSC during the encoding of virtual events by varying the level of visuomotor and perspectival congruency and collected ANC 1 week later. Our results show (1) that changes in functional connectivity strength between hippocampus and dPMC were associated with different levels of visuomotor and perspectival congruency. Moreover, premotor–hippocampal connectivity (2) modulated the relationship of SoA and SoO during encoding with later subjective ANC during reliving: Higher premotor–hippocampal connectivity was associated with a stronger BSC–ANC association, while lower connectivity led to a weaker association. These effects were only found in the left hemisphere (contralateral to hand movements) and absent for dPMC–hippocampus connectivity in the right hemisphere. These findings support the role of dPMC, a key BSC region, in ANC, linking subjective bodily experience during encoding to the subsequent subjective re-experiencing of past events.

### 4.3 Limitations

First, the present study reports a lateralized effect, where the functional connectivity in the left hemisphere (contralateral to the moving hand) between hippocampus and dPMC is significantly higher than the functional connectivity of the corresponding regions in the right hemisphere. This finding suggests that dPMC–hippocampus functional connectivity driving SoA–ANC coupling has a motor component. Future research should explore this hemispheric organization further by comparing functional connectivity in SoA modulations caused by right versus left upper limb movements. Second, our study focused on the premotor–hippocampal functional coupling during the encoding of an event. However, we do not rule out that such coupling might also exist during consolidation or retrieval phases, further impacting the association between BSC and ANC. We encourage future studies to investigate both the role of BSC state and the related premotor–hippocampal coupling during these periods. Additionally, we acknowledge that this study focused on a priori regions of interest (the hippocampus and dPMC) to investigate the association between ANC and BSC as these two brain regions were recently found to have a BSC-dependent coupled reinstatement during scene recognition task (Meyer, Gauthier, Stampacchia, et al., 2024). However, other regions may also contribute to this association. For instance, previous studies have implicated the angular gyrus in memory-related processes (Addis et al., 2004, 2012; Bréchet et al., 2019; Iriye et al., 2024; Iriye & St. Jacques, 2025). Given our limited sample size, we believe that a hypothesis-driven, ROI-based approach was the most appropriate for the current study. Nevertheless, future research should employ experimental designs with broader spatial coverage to better characterize the full network of brain regions involved in the link between ANC and BSC. Although our results demonstrate a link between ANC and SoA at encoding, the vertical movements performed by participants were not directly related to the objects in the scene. Future studies should, therefore, develop scenarios in which SoA (and BSC) is examined in the context of object-related movements, to better understand how such interactions influence ANC. However, we note that the majority of everyday movements are not directly linked to objects in the environment. Thus, the fact that we observed a relationship between SoA and ANC even with movements irrelevant to objects suggests that the premotor–hippocampal link may be even stronger when movements are directly related to objects. Finally, the results presented in this study are subject to a mathematical limitation. As noted in the section 2, while the significant findings suggest that hippocampal activity can account for dPMC activity, the low temporal resolution (TR = 1.5) prevents us from inferring the biological significance of this relationship. Consequently, these results should be interpreted cautiously, indicating significant co-activation between the hippocampus and dPMC, without implying a directional influence between the two regions.

## Supplementary Material

Supplementary Material

## Data Availability

Code and data necessary to reproduce the results reported in this paper are available on the OSF platform (https://osf.io/wkcnm/overview?view_only=1e099abe8bee43fc9716b9351385aa77). The access to the imaging data will require a formal data use agreement. Requests will be evaluated based on institutional and departmental policies to determine whether the data requested are subject to intellectual property or patient privacy obligations.

## References

[IMAG.a.1059-b1] Addis, D. R., Knapp, K., Roberts, R. P., & Schacter, D. L. (2012). Routes to the past: Neural substrates of direct and generative autobiographical memory retrieval. NeuroImage, 59, 2908–2922. 10.1016/j.neuroimage.2011.09.066 22001264 PMC3254828

[IMAG.a.1059-b2] Addis, D. R., Moscovitch, M., Crawley, A. P., & McAndrews, M. P. (2004). Recollective qualities modulate hippocampal activation during autobiographical memory retrieval. Hippocampus, 14, 752–762. 10.1002/hipo.10215 15318333

[IMAG.a.1059-b3] Bartsch, T., Döhring, J., Rohr, A., Jansen, O., & Deuschl, G. (2011). CA1 neurons in the human hippocampus are critical for autobiographical memory, mental time travel, and autonoetic consciousness. Proceedings of the National Academy of Sciences, 108, 17562–17567. 10.1073/pnas.1110266108 PMC3198338 21987814

[IMAG.a.1059-b4] Ben-Yakov, A., & Dudai, Y. (2011). Constructing realistic engrams: Poststimulus activity of hippocampus and dorsal striatum predicts subsequent episodic memory. Journal of Neuroscience, 31, 9032–9042. https://www.jneurosci.org/content/31/24/9032.short 21677186 10.1523/JNEUROSCI.0702-11.2011 PMC6622928

[IMAG.a.1059-b5] Bergouignan, L., Nyberg, L., & Ehrsson, H. H. (2014). Out-of-body–induced hippocampal amnesia. Proceedings of the National Academy of Sciences, 111, 4421–4426. 10.1073/pnas.1318801111 PMC3970513 24616529

[IMAG.a.1059-b6] Berna, F., Potheegadoo, J., Aouadi, I., Ricarte, J. J., Allé, M. C., Coutelle, R., Boyer, L., Cuervo-Lombard, C. V., & Danion, J.-M. (2015). A meta-analysis of autobiographical memory studies in schizophrenia spectrum disorder. Schizophrenia Bulletin, 42(1), 56–66. 10.1093/schbul/sbv099 26209548 PMC4681554

[IMAG.a.1059-b200] Blanke, O., Ortigue, S., Landis, T., & Seeck, M. (2002). Stimulating illusory own-body perceptions. Nature, 419, 269–270. 10.1038/419269a 12239558

[IMAG.a.1059-b7] Blanke, O., Slater, M., & Serino, A. (2015). Behavioral, neural, and computational principles of bodily self-consciousness. Neuron, 88, 145–166. 10.1016/j.neuron.2015.09.029 26447578

[IMAG.a.1059-b8] Bréchet, L., Grivaz, P., Gauthier, B., & Blanke, O. (2018). Common recruitment of angular gyrus in episodic autobiographical memory and bodily self-consciousness. Frontiers in Behavioral Neuroscience, 12, 270. 10.1101/345991 30487740 PMC6246737

[IMAG.a.1059-b9] Bréchet, L., Mange, R., Herbelin, B., Theillaud, Q., Gauthier, B., Serino, A., & Blanke, O. (2019). First-person view of one’s body in immersive virtual reality: Influence on episodic memory. PLoS One, 14, e0197763. 10.1371/journal.pone.0197763 30845269 PMC6405051

[IMAG.a.1059-b10] Bullington, J. (2009). Embodiment and chronic pain: Implications for rehabilitation practice. Health Care Analysis, 17, 100–109. 10.1007/s10728-008-0109-5 19184443

[IMAG.a.1059-b11] Conway, A., Conway, M., Aggleton, J., & Conway, M. A. (2001). Sensory–perceptual episodic memory and its context: Autobiographical memory. Philosophical Transactions of the Royal Society of London. Series B: Biological Sciences, 356, 1375–1384. 10.1098/rstb.2001.0940 11571029 PMC1088521

[IMAG.a.1059-b12] Corcoran, R., & Frith, C. D. (2003). Autobiographical memory and theory of mind: Evidence of a relationship in schizophrenia. Psychological Medicine, 33, 897–905. 10.1017/S0033291703007529 12877404

[IMAG.a.1059-b13] De Vignemont, F. (2011). A self for the body. Metaphilosophy, 42, 230–247. 10.1111/j.1467-9973.2011.01688.x

[IMAG.a.1059-b14] Debarba, H. G., Bovet, S., Salomon, R., Blanke, O., Herbelin, B., & Boulic, R. (2017). Characterizing first and third person viewpoints and their alternation for embodied interaction in virtual reality. PLoS One, 12, e0190109. 10.1371/journal.pone.0190109 29281736 PMC5744958

[IMAG.a.1059-b15] Dudai, Y., & Eisenberg, M. (2004). Rites of passage of the engram: Reconsolidation and the lingering consolidation hypothesis. Neuron, 44, 93–100. 10.1016/j.neuron.2004.09.003 15450162

[IMAG.a.1059-b16] Ehrsson, H. H. (2012). The concept of body ownership and its relation to multisensory integration. In Stein, B.E. (Ed.), The new handbook of multisensory processing (pp. 775–792). The MIT Press. 10.7551/mitpress/8466.003.0067

[IMAG.a.1059-b17] Ehrsson, H. H. (2007). The experimental induction of out-of-body experiences. Science, 317, 1048–1048. 10.1126/science.1142175 17717177

[IMAG.a.1059-b18] Fourneret, P., & Jeannerod, M. (1998). Limited conscious monitoring of motor performance in normal subjects. Neuropsychologia, 36, 1133–1140. 10.1016/S0028-3932(98)00006-2 9842759

[IMAG.a.1059-b19] Gardiner, A., Conway, M., Aggleton, J., & Gardiner, J. M. (2001). Episodic memory and autonoetic consciousness: A first–person approach. Philosophical Transactions of the Royal Society of London. Series B: Biological Sciences, 356, 1351–1361. 10.1098/rstb.2001.0955 11571027 PMC1088519

[IMAG.a.1059-b20] Gauthier, B., Bréchet, L., Lance, F., Mange, R., Herbelin, B., Faivre, N., Bolton, T. A. W., Ville, D. V. D., & Blanke, O. (2020). First-person body view modulates the neural substrates of episodic memory and autonoetic consciousness: A functional connectivity study. NeuroImage, 223, 117370. 10.1016/j.neuroimage.2020.117370 32931940

[IMAG.a.1059-b21] Girondini, M., Mariano, M., Stanco, G., Gallace, A., & Zapparoli, L. (2025). Human bodies in virtual worlds: A systematic review of implicit sense of agency and ownership measured in immersive virtual reality environments. Frontiers in Human Neuroscience, 19, 1553574. 10.3389/fnhum.2025.1553574 40852503 PMC12367803

[IMAG.a.1059-b22] Gomez, A., Rousset, S., & Charnallet, A. (2012). Spatial deficits in an amnesic patient with hippocampal damage: Questioning the multiple trace theory. Hippocampus, 22, 1313–1324. 10.1002/hipo.20968 21805527

[IMAG.a.1059-b23] Grivaz, P., Blanke, O., & Serino, A. (2017). Common and distinct brain regions processing multisensory bodily signals for peripersonal space and body ownership. NeuroImage, 147, 602–618. 10.1016/j.neuroimage.2016.12.052 28017920

[IMAG.a.1059-b24] Haggard, P. (2017). Sense of agency in the human brain. Nature Reviews Neuroscience, 18, 196–207. 10.1038/nrn.2017.14 28251993

[IMAG.a.1059-b25] Humphreys, G. F., Lambon Ralph, M. A., & Simons, J. S. (2021). A unifying account of angular gyrus contributions to episodic and semantic cognition. Trends in Neurosciences, 44, 452–463. 10.1016/j.tins.2021.01.006 33612312

[IMAG.a.1059-b26] Illman, N. A., Rathbone, C. J., Kemp, S., & Moulin, C. J. A. (2011). Autobiographical memory and the self in a case of transient epileptic amnesia. Epilepsy & Behavior, 21, 36–41. 10.1016/j.yebeh.2011.02.022 21482196

[IMAG.a.1059-b201] Ionta, S., Heydrich, L., Lenggenhager, B., Mouthon, M., Fornari, E., Chapuis, D., Gassert, R., & Blanke, O. (2011). Multisensory mechanisms in temporo-parietal cortex support self-location and first-person perspective. Neuron, 70(2), 363–374. 10.1016/j.neuron.2011.03.009 21521620

[IMAG.a.1059-b27] Irish, M., Lawlor, B. A., O'Mara, S. M., & Coen, R. F. (2008). Assessment of behavioural markers of autonoetic consciousness during episodic autobiographical memory retrieval: A preliminary analysis. Behavioral Neurology, 19, 3–6. https://content.iospress.com/articles/behavioural-neurology/ben00190 18413908 10.1155/2008/691925 PMC5452460

[IMAG.a.1059-b28] Irish, M., Lawlor, B. A., O’Mara, S. M., & Coen, R. F. (2011). Impaired capacity for autonoetic reliving during autobiographical event recall in mild Alzheimer’s disease. Cortex, 47, 236–249. 10.1016/j.cortex.2010.01.002 20153463

[IMAG.a.1059-b29] Iriye, H., Chancel, M., & Ehrsson, H. H. (2024). Sense of own body shapes neural processes of memory encoding and reinstatement. Cerebral Cortex, *34*, bhad443. 10.1093/cercor/bhad443 PMC10793569 38012107

[IMAG.a.1059-b30] Iriye, H., & Ehrsson, H. H. (2022). Perceptual illusion of body-ownership within an immersive realistic environment enhances memory accuracy and re-experiencing. iScience, 25, 103584. 10.1016/j.isci.2021.103584 35005534 PMC8717413

[IMAG.a.1059-b31] Iriye, H. M., & St. Jacques, P. L. (2025). An embodied perspective: Angular gyrus and precuneus decode selfhood in memories of naturalistic events. Imaging Neuroscience, 3, IMAG.a.93. 10.1162/IMAG.a.93 PMC12330842 40800785

[IMAG.a.1059-b32] Johnson, M. K., Foley, M. A., Suengas, A. G., & Raye, C. L. (1988). Phenomenal characteristics of memories for perceived and imagined autobiographical events. Journal of Experimental Psychology: General, 117, 371–376. 10.1037/0096-3445.117.4.371 2974863

[IMAG.a.1059-b33] Josselyn, S. A., Köhler, S., & Frankland, P. W. (2015). Finding the engram. Nature Reviews Neuroscience, 16, 521–534. 10.1038/nrn4000 26289572

[IMAG.a.1059-b202] Kaiser, H. F., & Rice, J. (1974). Little Jiffy, Mark IV. Educational and Psychological Measurement, 34(1), 111–117. 10.1177/001316447403400115

[IMAG.a.1059-b34] Kannape, O. A., & Blanke, O. (2013). Self in motion: Sensorimotor and cognitive mechanisms in gait agency. Journal of Neurophysiology, 110, 1837–1847. 10.1152/jn.01042.2012 23825398

[IMAG.a.1059-b35] Klein, S. B., Gangi, C. E., & Lax, M. L. (2011). Memory and self-knowledge in young adults with ADHD. Self and Identity, 10, 213–230. 10.1080/15298861003741604

[IMAG.a.1059-b36] Klein, S. B., German, T. P., Cosmides, L., & Gabriel, R. (2004). A theory of autobiographical memory: Necessary components and disorders resulting from their loss. Social Cognition, 22, 460–490. 10.1521/soco.22.5.460.50765

[IMAG.a.1059-b37] Klein, S. B., & Nichols, S. (2012). Memory and the sense of personal identity. Mind, 121, 677–702. 10.1093/mind/fzs080

[IMAG.a.1059-b38] Kokkinara, E., Kilteni, K., Blom, K. J., & Slater, M. (2016). First person perspective of seated participants over a walking virtual body leads to illusory agency over the walking. Scientific Reports, 6, 28879. 10.1038/srep28879 PMC4929480 27364767

[IMAG.a.1059-b39] Lavenex, P., & Amaral, D. G. (2000). Hippocampal-neocortical interaction: A hierarchy of associativity. Hippocampus, 10, 420–430. 10.1002/1098-1063(2000)10:4<420::AID-HIPO8>3.0.CO;2-5 10985281

[IMAG.a.1059-b40] Lenggenhager, B., Tadi, T., Metzinger, T., & Blanke, O. (2007). Video ergo sum: Manipulating bodily self-consciousness. Science, 317, 1096–1099. 10.1126/science.1143439 17717189

[IMAG.a.1059-b41] Lenormand, D., Mentec, I., Gaston-Bellegarde, A., Orriols, E., & Piolino, P. (2024). Decoding episodic autobiographical memory in naturalistic virtual reality. Scientific Reports, 14, 25639. 10.1038/s41598-024-76944-3 39463396 PMC11514229

[IMAG.a.1059-b204] Makowski, D. (2018). The psycho package: An efficient and publishing-oriented workflow for psychological science. Journal of Open Source Software, 3(22), 470. 10.21105/joss.00470

[IMAG.a.1059-b42] Markowitsch, H. J., & Staniloiu, A. (2011). Memory, autonoetic consciousness, and the self. Consciousness and Cognition, Brain and Self: Bridging the Gap, 20, 16–39. 10.1016/j.concog.2010.09.005 20951059

[IMAG.a.1059-b43] McCormick, C., St-Laurent, M., Ty, A., Valiante, T. A., & McAndrews, M. P. (2015). Functional and effective hippocampal–Neocortical connectivity during construction and elaboration of autobiographical memory retrieval. Cerebral Cortex, 25, 1297–1305. 10.1093/cercor/bht324 24275829 PMC4397572

[IMAG.a.1059-b44] Meyer, N. H., Babo-Rebelo, M., Potheegadoo, J., Duong Phan Thanh, L., Boscheron, J., Herbelin, B., Vuarnesson, L., Stampacchia, S., Toye, I. M., Esposito, F., Morais Lacerda, M., Trivier, A., Beanato, E., Alvarez, V., Bassolino, M., & Blanke, O. (2025). Bodily perception links memory and self: A case study of an amnesic patient. Cortex, 191, 245–265. 10.1016/j.cortex.2025.07.015 40907237

[IMAG.a.1059-b45] Meyer, N. H., Gauthier, B., Stampacchia, S., Boscheron, J., Babo-Rebelo, M., Potheegadoo, J., Herbelin, B., Lance, F., Alvarez, V., Franc, E., Esposito, F., Morais Lacerda, M., & Blanke, O. (2024). Embodiment in episodic memory through premotor-hippocampal coupling. Communications Biology, 7, 1–19. 10.1038/s42003-024-06757-7 39256570 PMC11387647

[IMAG.a.1059-b46] Meyer, N. H., Gauthier, B., Potheegadoo, J., Boscheron, J., Franc, E., Lance, F., & Blanke, O. (2024). Sense of agency during encoding predicts subjective reliving. eNeuro, 11, ENEURO.0256-24.2024. 10.1523/ENEURO.0256-24.2024 PMC11613308 39317465

[IMAG.a.1059-b47] Moscovitch, M., Cabeza, R., Winocur, G., & Nadel, L. (2016). Episodic memory and beyond: The hippocampus and neocortex in transformation. Annual Review of Psychology, 67, 105–134. 10.1146/annurev-psych-113011-143733 PMC5060006 26726963

[IMAG.a.1059-b48] Murre, J. M. J., Kristo, G., & Janssen, S. M. J. (2014). The effect of self-reported habitual sleep quality and sleep length on autobiographical memory. Memory, 22, 633–645. 10.1080/09658211.2013.811253 23815161

[IMAG.a.1059-b49] Nadel, L., Samsonovich, A., Ryan, L., & Moscovitch, M. (2000). Multiple trace theory of human memory: Computational, neuroimaging, and neuropsychological results. Hippocampus, 10, 352–368. 10.1002/1098-1063(2000)10:4<352::AID-HIPO2>3.0.CO;2-D 10985275

[IMAG.a.1059-b203] Nicholls, M. E. R., Thomas, N. A., Loetscher, T., & Grimshaw, G. M. (2013). The Flinders Handedness survey (FLANDERS): A brief measure of skilled hand preference. Cortex, 49(10), 2914–2926. 10.1016/j.cortex.2013.02.002 23498655

[IMAG.a.1059-b50] Noel, J.-P., Blanke, O., & Serino, A. (2018). From multisensory integration in peripersonal space to bodily self-consciousness: From statistical regularities to statistical inference. Annals of the New York Academy of Sciences, 1426, 146–165. 10.1111/nyas.13867 29876922

[IMAG.a.1059-b51] Padilla-Castañeda, M. A., Frisoli, A., Pabon, S., & Bergamasco, M. (2014). The modulation of ownership and agency in the virtual hand illusion under visuotactile and visuomotor sensory feedback. Presence, 23, 209–225. 10.1162/PRES_a_00181

[IMAG.a.1059-b52] Park, H. D., & Blanke, O. (2019). Coupling inner and outer body for self-consciousness. Trends in Cognitive Sciences, 23, 377–388. 10.1016/j.tics.2019.02.002 30826212

[IMAG.a.1059-b53] Piolino, P., Desgranges, B., Belliard, S., Matuszewski, V., Lalevée, C., De La Sayette, V., & Eustache, F. (2003). Autobiographical memory and autonoetic consciousness: Triple dissociation in neurodegenerative diseases. Brain, 126, 2203–2219. 10.1093/brain/awg222 12821510

[IMAG.a.1059-b54] Piolino, P., Desgranges, B., Benali, K., & Eustache, F. (2002). Episodic and semantic remote autobiographical memory in ageing. Memory, 10, 239–257. 10.1080/09658210143000353 12097209

[IMAG.a.1059-b55] Piolino, P., Desgranges, B., Clarys, D., Guillery-Girard, B., Taconnat, L., Isingrini, M., & Eustache, F. (2006). Autobiographical memory, autonoetic consciousness, and self-perspective in aging. Psychology and Aging, 21, 510–525. 10.1037/0882-7974.21.3.510 16953713

[IMAG.a.1059-b56] Potheegadoo, J., Berna, F., Cuervo-Lombard, C., & Danion, J.-M. (2013). Field visual perspective during autobiographical memory recall is less frequent among patients with schizophrenia. Schizophrenia Research, 150, 88–92. 10.1016/j.schres.2013.07.035 23932447

[IMAG.a.1059-b57] Potheegadoo, J., Cuervo-Lombard, C., Berna, F., & Danion, J.-M. (2012). Distorted perception of the subjective temporal distance of autobiographical events in patients with schizophrenia. Consciousness and Cognition, Beyond the Comparator Model, 21, 90–99. 10.1016/j.concog.2011.09.012 21993451

[IMAG.a.1059-b58] Rimmele, U., Besedovsky, L., Lange, T., & Born, J. (2015). Emotional memory can be persistently weakened by suppressing cortisol during retrieval. Neurobiology of Learning and Memory, 119, 102–107. 10.1016/j.nlm.2015.01.010 25680817

[IMAG.a.1059-b205] R Core Team. (2022). R: A language and environment for statistical computing. R Foundation for Statistical Computing, Vienna, Austria. https://www.R-project.org/

[IMAG.a.1059-b59] Riutort, M., Cuervo, C., Danion, J.-M., Peretti, C. S., & Salamé, P. (2003). Reduced levels of specific autobiographical memories in schizophrenia. Psychiatry Research, 117, 35–45. 10.1016/S0165-1781(02)00317-7 12581819

[IMAG.a.1059-b250] RStudio Team. (2022). RStudio: Integrated Development Environment for R. RStudio, PBC, Boston, MA. http://www.rstudio.com/

[IMAG.a.1059-b60] Ruby, P., & Decety, J. (2001). Effect of subjective perspective taking during simulation of action: A PET investigation of agency. Nature Neuroscience, 4, 546–550. 10.1038/87510 11319565

[IMAG.a.1059-b61] Salomon, R., Fernandez, N. B., van Elk, M., Vachicouras, N., Sabatier, F., Tychinskaya, A., Llobera, J., & Blanke, O. (2016). Changing motor perception by sensorimotor conflicts and body ownership. Scientific Reports, 6, 25847. 10.1038/srep25847 27225834 PMC4881011

[IMAG.a.1059-b62] Scattolin, M., Panasiti, M. S., Villa, R., & Aglioti, S. M. (2022). Reduced ownership over a virtual body modulates dishonesty. iScience, 25, 104320. 10.1016/j.isci.2022.104320 35602961 PMC9118670

[IMAG.a.1059-b63] Seghezzi, S., Giannini, G., & Zapparoli, L. (2019). Neurofunctional correlates of body-ownership and sense of agency: A meta-analytical account of self-consciousness. Cortex, 121, 169–178. 10.1016/j.cortex.2019.08.018 31629195

[IMAG.a.1059-b64] Sekeres, M. J., Winocur, G., & Moscovitch, M. (2018). The hippocampus and related neocortical structures in memory transformation. Neuroscience Letters, New Perspectives on the Hippocampus and Memory, 680, 39–53. 10.1016/j.neulet.2018.05.006 29733974

[IMAG.a.1059-b65] Shimamura, A. P. (2010). Hierarchical relational binding in the medial temporal lobe: The strong get stronger. Hippocampus, 20, 1206–1216. 10.1002/hipo.20856 20824723

[IMAG.a.1059-b66] Squire, L. R. (1992). Memory and the hippocampus: A synthesis from findings with rats, monkeys, and humans. Psychological Review, 99, 195–231. 10.1037/0033-295X.99.2.195 1594723

[IMAG.a.1059-b67] Squire, L. R., Genzel, L., Wixted, J. T., & Morris, R. G. (2015). Memory consolidation. Cold Spring Harbor Perspectives in Biology, 7, a021766. 10.1101/cshperspect.a021766 26238360 PMC4526749

[IMAG.a.1059-b68] Svenaeus, F. (2015). The phenomenology of chronic pain: Embodiment and alienation. Continental Philosophy Review, 48, 107–122. 10.1007/s11007-015-9325-5

[IMAG.a.1059-b69] Tanaka, K. Z., & McHugh, T. J. (2018). The hippocampal engram as a memory index. Journal of Experimental Neuroscience, 12, 1179069518815942. 10.1177/1179069518815942 30546263 PMC6287299

[IMAG.a.1059-b70] Tsakiris, M. (2010). My body in the brain: A neurocognitive model of body-ownership. Neuropsychologia, The Sense of Body, 48, 703–712. 10.1016/j.neuropsychologia.2009.09.034 19819247

[IMAG.a.1059-b71] Tsakiris, M., Longo, M. R., & Haggard, P. (2010). Having a body versus moving your body: Neural signatures of agency and body-ownership. Neuropsychologia, 48, 2740–2749. 10.1016/j.neuropsychologia.2010.05.021 20510255

[IMAG.a.1059-b72] Tulving, E. (1985). Memory and consciousness. Canadian Psychology/Psychologie Canadienne, 26, 1–12. 10.1037/h0080017

[IMAG.a.1059-b73] Weijs, M. L., Macartney, E., Daum, M. M., & Lenggenhager, B. (2021). Development of the bodily self: Effects of visuomotor synchrony and visual appearance on virtual embodiment in children and adults. Journal of Experimental Child Psychology, 210, 105200. 10.1016/j.jecp.2021.105200 34116407

[IMAG.a.1059-b74] Wheeler, M. A., Stuss, D. T., & Tulving, E. (1997). Toward a theory of episodic memory: The frontal lobes and autonoetic consciousness. Psychological Bulletin, 121, 331–354. 10.1037/0033-2909.121.3.331 9136640

[IMAG.a.1059-b75] Zeiler, K. (2010). A phenomenological analysis of bodily self-awareness in the experience of pain and pleasure: On dys-appearance and eu-appearance. Medicine Health Care and Philosophy, 13, 333–342. 10.1007/s11019-010-9237-4 20162369

