# Supplementary material for: Subjective reliving of past events is modulated by premotor–hippocampal coupling and bodily self-consciousness during event encoding
Source: Imaging Neurosci (Camb). 2025 Dec 15;3:IMAG.a.1059. doi: 10.1162/IMAG.a.1059 (PMC12706697; doi:10.1162/IMAG.a.1059)
Supplement: Supplementary Material [file IMAG.a.1059_supp.pdf]

Subjective reliving of past events is modulated by premotor-hippocampal coupling and bodily self-consciousness during event encoding

Authors

Nathalie Heidi Meyer[1], Lucas Burget[1], Baptiste Gauthier[1,2], Jevita Potheegadoo[1], Juliette Boscheron[1], Olaf Blanke[1,3]

Affiliations

[1] Laboratory of Cognitive Neuroscience, Neuro-X Institute, Faculty of Life Sciences, Ecole Polytechnique Fédérale de Lausanne, 1202 Geneva, Switzerland

[2] Clinical Research Unit, Neuchâtel Hospital Network, 2000 Neuchâtel, Switzerland

[3] Department of Clinical Neurosciences, University Hospital Geneva, Rue Micheli-du-Crest 24, 1205, Geneva Switzerland

Supplementary materials

Supplementary Tables

**1.**	**SYNCH1PP MRI/Behavioral**	**ASYNCH1PP MRI/Behavioral**	**ASYNCH3PP MRI/Behavioral**
**SoA**	0.61±0.23/0.66±0.24	0.54±0.3/0.61±0.26	0.5±0.3/0.59±0.25
**SoO**	0.38±0.22/0.53±0.27	0.41±0.3/0.5±0.3	0.3±0.27/0.4±0.27
**Mean Control**	0.15±0.17/0.12±0.14	0.6±0.21/0.12±0.13	0.12± 0.14/0.1±0.11

Supplementary table 1: mean and standard deviation of the different BSC ratings under the three conditions for the MRI experiment and the behavioral experiment. SoA = sense of agency, SoO = sense of ownership, nMRI = 24, nBehavioral = 49.

	estimate	t-value	p-value
Intercept	-0.5	-1.76	0.08
ASYNCH1PP	0.62	2.42	0.02*
ASYNCH3PP	0.87	3.37	0.001**
SoA	0.61	1.58	0.11
Experiment Behavioral	-0.13	-0.64	0.53
ASYNCH1PP*SoA	-0.69	-1.77	0.08
ASYNCH3PP*SoA	-1.14	-2.91	0.004**
ASYNCH3PP* SoA (compared with ASYNCH1PP)	-0.45	-1.26	0.21

Supplementary table 2: ANC-1 ~SoA * Conditions + Experiment+ (1| Participants)

	estimate	t-value	p-value
Intercept	-0.03	-0.12	0.9
ASYNCH1PP	-0.11	-0.51	0.61
ASYNCH3PP	-0.23	-1.03	0.3
SoA	-0.13	-0.37	0.7
Experiment Behavioral	0.29	1.7	0.09
ASYNCH1PP*SoA	0.23	0.68	0.5
ASYNCH3PP*SoA	0.33	0.99	0.32
ASYNCH3PP * SoA (compared with ASYNCH1PP)	0.11	0.35	0.73

Supplementary table 3: ANC-2 ~SoA * Conditions + Experiment+ (1| Participants)

	estimate	t-value	p-value
Intercept	-0.05	-0.24	0.81
ASYNCH1PP	-0.02	0.07	0.95
ASYNCH3PP	0.04	0.25	0.8
SoA	0.14	0.5	0.62
Experiment Behavioral	-0.25	-1.61	0.11

ASYNCH1PP*SoA	0.007	0.027	0.98
ASYNCH3PP*SoA	-0.24	-0.85	0.4
ASYNCH3PP * SoA (compared with ASYNCH1PP)	-0.25	-0.96	0.34

Supplementary table 4: ANC-3 ~SoA * Conditions + Experiment+ (1| Participants)

	estimate	t-value	p-value
Intercept	-0.28	-1.32	0.19
ASYNCH1PP	0.28	1.43	0.15
ASYNCH3PP	0.43	2.24	0.03*
SoO	0.35	1.04	0.3
Experiment Behavioral	-0.1	-0.51	0.61
ASYNCH1PP*SoO	-0.19	-0.53	0.6
ASYNCH3PP*SoO	-0.56	-1.48	0.14
ASYNCH3PP * SoO (compared with ASYNCH1PP)	-0.37	-1.06	0.29

Supplementary table 5: ANC-1 ~SoO * Conditions + Experiment+ (1| Participants)

	estimate	t-value	p-value
Intercept	0.002	0.016	0.99
ASYNCH1PP	-0.14	-0.82	0.41
ASYNCH3PP	-0.18	-1.089	0.28
SoO	-0.22	-0.78	0.43
Experiment Behavioral	0.28	0.621	0.11
ASYNCH1PP*SoO	0.34	1.11	0.27
ASYNCH3PP*SoO	0.33	1.0	0.32
ASYNCH3PP * SoO (compared with ASYNCH1PP)	-0.01	-0.05	0.96

Supplementary table 6: ANC-2 ~SoO * Conditions + Experiment+ (1| Participants)

	estimate	t-value	p-value
Intercept	-0.2	-1.3	0.2
ASYNCH1PP	0.13	0.92	0.36
ASYNCH3PP	0.06	0.47	0.64
SoO	0.49	1.99	0.048*
Experiment Behavioral	-0.22	-1.4	0.17
ASYNCH1PP*SoO	-0.24	-0.96	0.34
ASYNCH3PP*SoO	-0.28	-1.06	0.29

ASYNCH3PP * SoO (compared with ASYNCH1PP)	-0.04	0.17	0.87

Supplementary table 7: ANC-3 ~SoO * Conditions + Experiment+ (1| Participants)

	estimate	t-value	p-value
Intercept	0.29	0.86	0.4
Functional connectivity	0.22	0.42	0.68
SoA	-0.73	-1.49	0.14
Functional connectivity * SoA	-0.17	-0.22	0.83

Supplementary table 8: ANC-1 ~SoA * Functional connectivity (left dPMC$\rightarrow$ left Hippocampus) + (1| Participants)

	estimate	t-value	p-value
Intercept	0.34	1.05	0.3
Functional connectivity	-0.008	-0.007	0.99
SoA	-0.79	-1.64	0.11
Functional connectivity * SoA	0.1	0.062	0.95

Supplementary table 9: ANC-1 ~SoA * Functional connectivity (left Hippocampus$\rightarrow$ dPMC) + (1| Participants)

	estimate	t-value	p-value
Intercept	0.04	0.15	0.88
Functional connectivity	-0.63	-1.41	0.16
SoA	0.27	0.66	0.51
Functional connectivity * SoA	0.96	1.44	0.16

Supplementary table 10: ANC-2 ~SoA * Functional connectivity (left dPMC$\rightarrow$ left Hippocampus)+ (1| Participants)

	estimate	t-value	p-value
Intercept	0.2	0.79	0.43
Functional connectivity	-2.88	-2.99	0.004**
SoA	0.08	0.19	0.84
Functional connectivity * SoA	3.78	2.71	0.008**

Supplementary table 11: ANC-2 ~SoA * Functional connectivity (left Hippocampus$\rightarrow$ dPMC) + (1| Participants)

	estimate	t-value	p-value

Intercept	-0.28	-1.16	0.25
Functional connectivity	0.09	0.24	0.81
SoA	0.09	0.26	0.79
Functional connectivity * SoA	-0.43	-0.76	0.45

Supplementary table 12: ANC-3 ~SoA * Functional connectivity (left dPMC→ left Hippocampus)+ (1| Participants)

	estimate	t-value	p-value
Intercept	-0.29	-1.23	0.22
Functional connectivity	0.19	0.23	0.82
SoA	0.11	0.32	0.75
Functional connectivity * SoA	-1.04	-0.86	0.39

Supplementary table 13: ANC-3 ~SoA * Functional connectivity (left Hippocampus→ dPMC) + (1| Participants)

	estimate	t-value	p-value
Intercept	0.05	0.2	0.85
Functional connectivity	0.15	0.41	0.68
SoO	-0.43	-0.94	0.35
Functional connectivity * SoO	-0.15	-0.21	0.83

Supplementary table 14: ANC-1 ~SoO * Functional connectivity (left dPMC→ left Hippocampus)+ (1| Participants)

	estimate	t-value	p-value
Intercept	0.07	0.34	0.73
Functional connectivity	0.08	0.09	0.93
SoO	-0.45	-1.06	0.29
Functional connectivity * SoO	-0.34	-0.19	0.85

Supplementary table 15: ANC-1 ~SoO * Functional connectivity (left Hippocampus →left dPMC)+ (1| Participants)

	estimate	t-value	p-value
Intercept	0.13	0.66	0.51
Functional connectivity	-0.31	-1.03	0.31
SoO	0.12	0.3	0.76
Functional connectivity * SoO	0.63	1.05	0.3

Supplementary table 16: ANC-2 ~SoO * Functional connectivity (left dPMC→ left Hippocampus)+ (1| Participants)

	estimate	t-value	p-value
Intercept	0.23	1.24	0.22
Functional connectivity	-2.1	-2.7	0.009**
SoO	-0.007	-0.02	0.98
Functional connectivity * SoO	3.56	2.4	0.02*

Supplementary table 17: ANC-2 ~SoO * Functional connectivity (left Hippocampus →left dPMC)+ (1| Participants)

	estimate	t-value	p-value
Intercept	-0.41	-2.41	0.02*
Functional connectivity	-0.08	-0.31	0.75
SoO	0.54	1.66	0.1
Functional connectivity * SoO	-0.2	-0.43	0.67

Supplementary table 18: ANC-3 ~SoO * Functional connectivity (left dPMC→ left Hippocampus)+ (1| Participants)

	estimate	t-value	p-value
Intercept	-0.43	-2.6	0.013*
Functional connectivity	0.04	0.07	0.95
SoO	0.57	-1.88	0.07
Functional connectivity * SoO	-1.082	-0.883	0.38

Supplementary table 19: ANC-3 ~SoO * Functional connectivity (left Hippocampus →left dPMC)+ (1| Participants)

	estimate	t-value	p-value
Intercept	0.38	1.28	0.013*
Functional connectivity	1.16	0.06	0.95
SoA	-0.83	-1.88	0.07
Functional connectivity * SoA	-0.45	-0.883	0.38

Supplementary table 20: ANC-1 ~SoA * Functional connectivity (right dPMC→ right Hippocampus)+ (1| Participants)

	estimate	t-value	p-value
Intercept	0.34	1.16	0.25
Functional connectivity	1.1	0.97	0.33
SoA	-0.76	-1.75	0.09
Functional connectivity * SoA	-0.37	-0.2	0.84

Supplementary table 21: ANC-1 ~SoA * Functional connectivity (right Hippocampus →right dPMC)+ (1| Participants)

	estimate	t-value	p-value
Intercept	-0.17	-0.68	0.5
Functional connectivity	-0.3	-0.69	0.49
SoA	0.57	1.54	0.13
Functional connectivity * SoA	0.325	0.49	0.63

Supplementary table 22: ANC-2 ~SoA * Functional connectivity (right dPMC→ right Hippocampus)+ (1| Participants)

	estimate	t-value	p-value
Intercept	-0.16	-0.64	0.53
Functional connectivity	-0.26	-0.26	0.8
SoA	0.57	1.51	0.14
Functional connectivity * SoA	0.18	0.11	0.92

Supplementary table 23: ANC-2 ~SoA * Functional connectivity (right Hippocampus →right dPMC)+ (1| Participants)

	estimate	t-value	p-value
Intercept	-0.24	-1.15	0.26
Functional connectivity	0.29	0.82	0.42
SoA	0.00001	0.000001	0.99
Functional connectivity * SoA	-0.45	-0.83	0.41

Supplementary table 24: ANC-3 ~SoA * Functional connectivity (right dPMC→ right Hippocampus)+ (1| Participants)

	estimate	t-value	p-value
Intercept	-0.25	-1.18	0.24
Functional connectivity	0.39	0.46	0.65
SoA	0.01	0.036	0.97
Functional connectivity * SoA	-0.58	-0.41	0.68

Supplementary table 25: ANC-3 ~SoA * Functional connectivity (right Hippocampus →right dPMC)+ (1| Participants)

	estimate	t-value	p-value
Intercept	0.09	0.43	0.67
Functional connectivity	0.43	1.15	0.25
SoO	-0.46	-1.14	0.26
Functional connectivity * SoO	-0.3	0.41	0.68

Supplementary table 26: ANC-1 ~SoO * Functional connectivity (right dPMC→ right Hippocampus)+ (1| Participants)

	estimate	t-value	p-value
Intercept	0.08	0.4	0.69
Functional connectivity	0.86	1.02	0.31
SoO	-0.45	-1.13	0.26
Functional connectivity * SoO	0.05	0.026	0.99

Supplementary table 27: ANC-1 ~SoO * Functional connectivity (right Hippocampus →right dPMC)+ (1| Participants)

	estimate	t-value	p-value
Intercept	0.03	0.15	0.88
Functional connectivity	-0.29	-0.88	0.38
SoO	0.34	0.97	0.34
Functional connectivity * SoO	0.43	0.67	0.51

Supplementary table 28: ANC-2 ~SoO * Functional connectivity (right dPMC→ right Hippocampus)+ (1| Participants)

	estimate	t-value	p-value
Intercept	0.03	0.19	0.85
Functional connectivity	-0.38	-0.5	0.62
SoO	0.32	0.94	0.35
Functional connectivity * SoO	0.54	0.31	0.76

Supplementary table 29: ANC-2 ~SoO * Functional connectivity (right Hippocampus →right dPMC)+ (1| Participants)

	estimate	t-value	p-value
Intercept	-0.41	-2.62	0.012*
Functional connectivity	0.29	1.14	0.26
SoO	0.46	1.64	0.11
Functional connectivity * SoO	-0.58	-1.16	0.25

Supplementary table 30: ANC-3 ~SoO * Functional connectivity (right dPMC→ right Hippocampus)+ (1| Participants)

	estimate	t-value	p-value
Intercept	-0.42	-2.69	0.01*
Functional connectivity	0.52	0.87	0.39
SoO	0.48	1.68	0.1
Functional connectivity * SoO	-1.19	-0.87	0.39

Supplementary table 31: ANC-3 ~SoO * Functional connectivity (right Hippocampus →right dPMC)+ (1| Participants)

2. Supplementary Figures

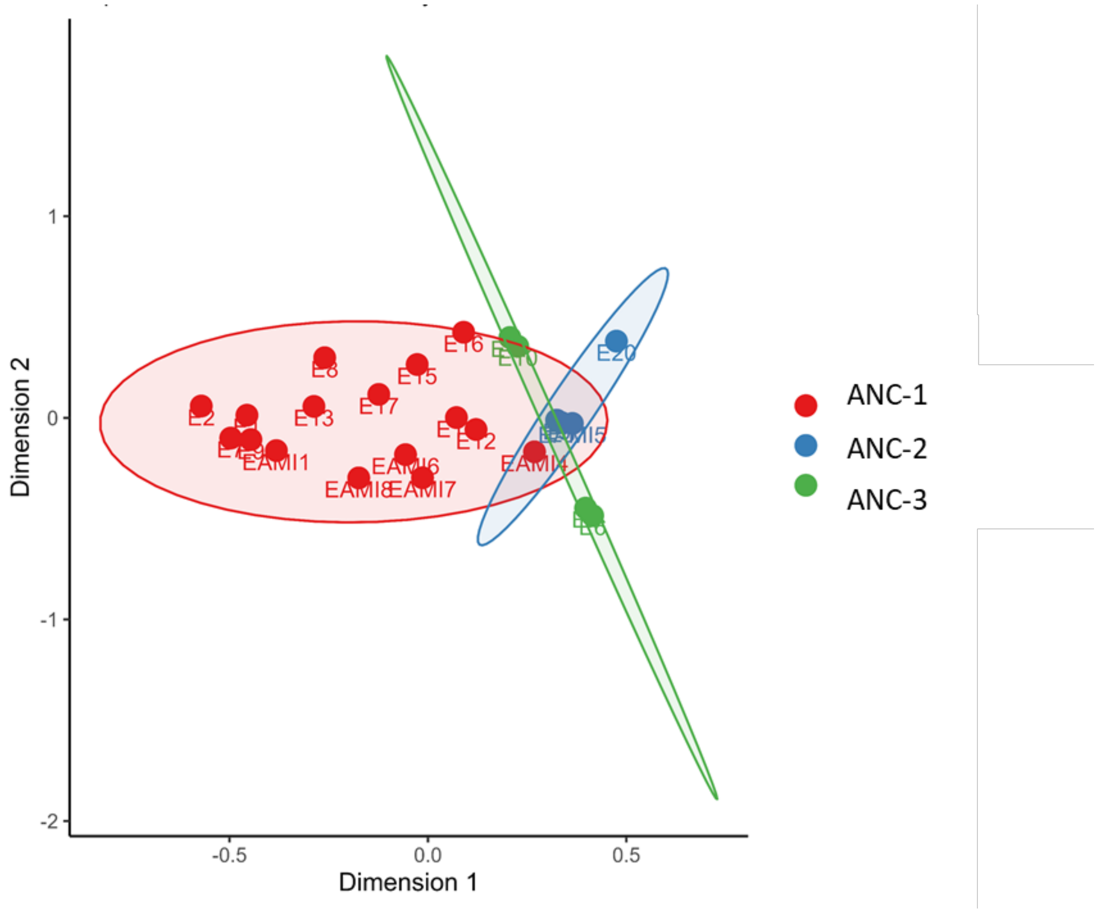

Supplementary Figure 1: Results of item clustering after the factor analysis.
